# “Dual Anta-Inhibitors” of the A_2A_ Adenosine Receptor and Casein Kinase CK1delta: Synthesis, Biological Evaluation, and Molecular Modeling Studies

**DOI:** 10.3390/ph16020167

**Published:** 2023-01-23

**Authors:** Andrea Spinaci, Michela Buccioni, Daniela Catarzi, Chang Cui, Vittoria Colotta, Diego Dal Ben, Eleonora Cescon, Beatrice Francucci, Ilenia Grieco, Catia Lambertucci, Gabriella Marucci, Davide Bassani, Matteo Pavan, Flavia Varano, Stephanie Federico, Giampiero Spalluto, Stefano Moro, Rosaria Volpini

**Affiliations:** 1 Medicinal Chemistry Unit, School of Pharmacy, University of Camerino, Via Madonna delle Carceri, 62032 Camerino, Italy; 2 Area del Farmaco e Salute del Bambino, Sezione di Farmaceutica e Nutraceutica, Dipartimento di Neuroscienze, Psicologia, Università degli Studi di Firenze, Via Ugo Schiff, 6, Sesto Fiorentino, 50019 Florence, Italy; 3 Department of Chemical and Pharmaceutical Sciences, University of Trieste, Via Licio Giorgieri 1, 34127 Trieste, Italy; 4 Molecular Modeling Section (MMS), Department of Pharmaceutical and Pharmacological Sciences, University of Padova, via Marzolo 5, 35131 Padova, Italy

**Keywords:** “dual anta-inhibitors”, CK1δ inhibitors, A_2A_ adenosine receptor antagonists, molecular modeling, computational study

## Abstract

Based on a screening of a chemical library of A_2A_ adenosine receptor (AR) antagonists, a series of di- and tri-substituted adenine derivatives were synthesized and tested for their ability to inhibit the activity of the enzyme casein kinase 1 delta (CK1δ) and to bind adenosine receptors (ARs). Some derivatives, here called “dual anta-inhibitors”, demonstrated good CK1δ inhibitory activity combined with a high binding affinity, especially for the A_2A_AR. The *N*^6^-methyl-(2-benzimidazolyl)-2-dimethyamino-9-cyclopentyladenine (**17**, IC_50_ = 0.59 μM and KiA_2A_ = 0.076 μM) showed the best balance of A_2A_AR affinity and CK1δ inhibitory activity. Computational studies were performed to simulate, at the molecular level, the protein–ligand interactions involving the compounds of our series. Hence, the dual anta-inhibitor **17** could be considered the lead compound of new therapeutic agents endowed with synergistic effects for the treatment of chronic neurodegenerative and cancer diseases.

## 1. Introduction

Casein kinases are highly conserved members of the serine/threonine protein kinases family, which phosphorylate specific proteins, utilizing ATP as a source of phosphate. They are present in all eukaryotes and regulate a wide range of cellular functions [1]. To date, seven isoforms of CK1 kinases have been characterized in mammals (α, β, γ1-3, δ, and ɛ) and six of them, and their various splice variants, have been found in humans, in which the β isoform is lacking [2]. All these isoforms are characterized by a highly conserved catalytic domain. In particular, the CK1δ and CK1ε isoforms show the highest conservation, with a similarity of >98% in terms of the amino acids in the catalytic domain [3]. The CK1δ isoform phosphorylates different substrates to play key pivotal roles in a number of pathophysiological processes such as circadian sleep disorder, neurodegenerative diseases, DNA repair, cell cycle progression, cancer, inflammation, cytokinesis, differentiation, and apoptosis. Over the past twenty years, several studies demonstrated its implication in many different cellular signal transduction pathways and the potential therapeutic use of its inhibitors in cancer and neurodegenerative diseases [4]. However, due to the similarities of the different CK1 isoforms, the development of specific inhibitors is still a challenge [5,6].

**Figure 1:**
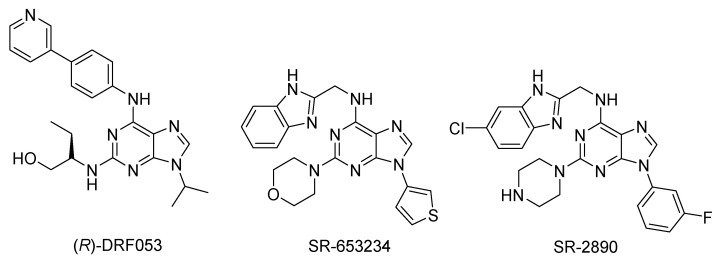
Structures of adenine-based CK1 δ inhibitors.

CK1δ inhibitors belong to different chemical classes and among them are adenine derivatives, which have mostly been found from HTS campaigns as ATP competitive inhibitors. Among them, the roscovitine derivative (*R*)-DRF053 (Figure 1), an adenine based inhibitor trisubstituted at the 2-, *N*^6^-, and 9-positions resulted in a potent CK1δ/ε inhibitor with an IC_50_ value of 14 nM, that was also able to inhibit CDK5 (IC_50_ = 80 nM) and Aβ production [7,8], thus, behaving as a multitarget agent. Other trisubstituted adenine derivatives, bearing a methylbenzimidazole moiety at the *N*^6^-position, are a series of Wee1 degrader inhibitors, such as SR-653234 (Figure 1), which resulted in a selective CK1δ/ε inhibitor with an IC_50_ of 160 nM and 540 nM, respectively [9,10]. Since the thiophene group was supposed to produce highly reactive metabolic products, and due to the low pharmacokinetic properties of this derivative, analogues with a fluorophenyl ring in the adenine 9-positon and another hydrophilic group at the 2-positon were synthesized, such as SR-2890 bearing a piperazine ring at the adenine 2-positon [9]. This compound showed an IC_50_ value of 4 nM as a CK1δ/ε inhibitor and exhibited breast cancer antitumor activity targeting the Wnt/β catenin signal [11].

Another target that seems to play a crucial role in neuroinflammatory processes, neurodegenerative disorders, and cancer is the adenosine A_2A_ receptor (A_2A_AR), activated by the naturally occurring nucleoside adenosine (Ado) [12,13,14]. ARs belong to the superfamily of G protein coupled receptors (GPCRs) and, to date, four subtypes named A_1_, A_2A_, A_2B_, and A_3_ have been cloned and characterized. They are widespread in the central nervous system and in the periphery, and are coupled to inhibitory G proteins (A_1_ and A_3_ ARs) and stimulatory G proteins (A_2A_ and A_2B_ ARs) [15]. In particular, the A_2A_ subtype is localized in the basal ganglia where it is also co-expressed with the dopamine D2 receptor and its blockade enhances D2 receptor function [16]. For this reason, A_2A_AR antagonists emerged as a nondopaminergic therapy for Parkinson’s disease (PD) [17] leading to the commercialization of the A_2A_AR antagonist istradefylline as an adjunctive therapy for the treatment of patients suffering with this pathology [18].

On the other hand, the A_2A_AR blockade has been extensively demonstrated to produce anti-inflammatory effects, attributed to the receptors expressed by glial cells, microglia and astrocytes. Hence, antagonists of this receptor are able to reduce astroglial function during neuroinflammatory processes leading to a robust neuroprotective effect in various neurodegenerative conditions [19,20,21,22].

In recent years, A_2A_ARs have also became an attractive target for cancer, due to their immunosuppressive activities induced by Ado in the tumor microenvironment (TME). So, A_2A_AR antagonists, by counteracting the effects of Ado, exert an immune enhancing activity [23].

Thus, the availability of molecules able to inhibit the enzyme isoform CK1δ and to antagonize the A_2A_AR could generate new therapeutic agents endowed with synergistic effects for the treatment of chronic neurodegenerative and cancer diseases.

With this concept in mind, and in the search for new CK1δ inhibitors, we selected and tested a series of adenine derivatives available in our chemical library and previously designed and synthesized as A_2A_AR antagonists [24,25,26]. These compounds were tested on truncated CK1δ enzyme, which lacks the autoregulatory inhibitory portion present in the C-terminal domain.

**Figure 2:**
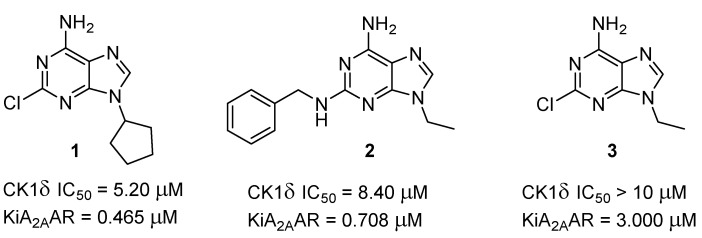
Structures and IC_50_ values of adenosine receptor antagonists endowed with CK1δ inhibitory activity.

**Figure 3:**
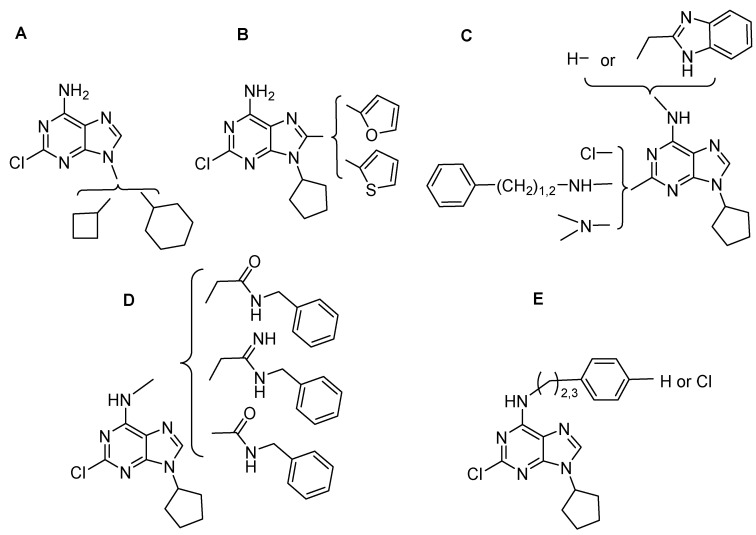
Designed molecules. Panels (**A**–**E**) represent the several modifications introduced at different positions of adenine.

Among the tested compounds, we found that the 2-chloro-9-cyclopentyladenine (**1**, KiA_2A_AR = 0.465 μM, Figure 2) [27] was able to inhibit the enzyme, leaving a residual activity of 0.7% at a concentration of 40 μM, while the residual activity was 13% when it was tested at 10 μM. The calculated IC_50_ for this compound was 5.20 μM. The other two adenine derivatives, endowed with CK1δ inhibitory activity, were the 9-ethyl-2-phenetylaminoadenine (**2**, KiA_2A_AR = 0.708 μM) [28] and its 2-chloro derivative **3** (KiA_2A_AR = 3.000 μM Figure 2) [29]. We noted that **2** (IC_50_ = 8.4 μM) was found to leave a residual activity of 5.5% and 39% at 40 μM and 10 μM, respectively, while, at the same concentrations, **3** was able to leave a residual activity of 41% and 71%, thus, its IC_50_ was estimated as > 10 μM. Starting from these observations, we selected the 2-chloro-9-cyclopentyladenine (**1**) as a starting molecule for further modification. Hence, as a first strategy and in order to assess the importance of the **1** cyclopentyl ring at the 9-position, we designed and synthesized analogues in which the cyclopentyl ring was replaced with a cyclobutyl or a cyclohexyl group (Figure 3A). Since these modifications did not lead to a significative improvement of the inhibitory activity, we investigated the influence of aromatic substituents in the 8-position of **1** by the introduction of a furyl and a thienyl ring (Figure 3B). This choice was guided by the fact that adenine derivatives substituted in the 8-position with such groups behave as potent A_2A_AR antagonists [30]. Then, taking into account the inhibitory activity of **2**, we replaced the 2-chlorine atom of **1** with a benzylamino group. This substituent was also length increased and simplified by a replacement with a phenethylamino and a dimethylamino group, respectively, to give the compounds of Figure 3C. With the aim of investigating the *N*^6^-substitution on these derivatives, we introduced, in that position, a methyl-(2-benzimidazolyl) ring, which was previously reported to enhance the CK1δ inhibitory activity of adenine derivatives [9].

Furthermore, in order to verify the importance of the methyl-(2-benzimidazolyl) ring for the inhibitory activity, in a second approach, we tried to simplify this substituent by replacing it with arylalkyl chains containing or not amido/imido functions (Figure 3D,E).

The newly synthesized compounds were tested for their ability to inhibit the CK1δ enzyme, as well as in binding assays and functional studies at ARs. Finally, a computational analysis to characterize, at the molecular level, the protein –ligand interactions involving the newly synthesized compounds, was performed.

## 2. Results and Discussion

### 2.1. Chemistry

The structure and physical-chemical properties of compound **1** are reported in the literature [27], though the synthetic procedure to obtain it was not already described to the best of our knowledge.

**Scheme 1:**
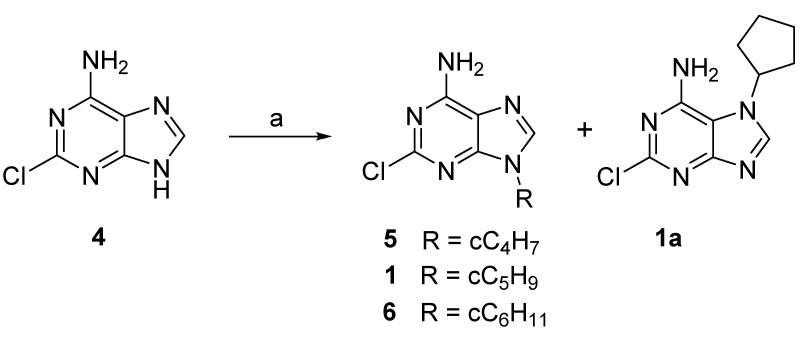
Reagents and conditions: a—K_2_CO_3_, cycloalkyl halide, r.t., 2 to 5 days.

The synthesis of the 2-chloro-9-cyclopentyladenine **1** was performed starting from the 2-chloroadenine (**4**) [31] that was reacted with bromo cyclopentane giving the formation of N-9 and N-7 isomers (**1** and **1a**, respectively, Scheme 1). It is well described in the literature that the alkylation of adenine or adenine derivatives leads to N-9 and N-7 as sites of alkylation with the N-9 isomer preferentially formed. Furthermore, in the normal phase TLC, the retention factors (Rfs) of the N-9 isomers are always higher than those of the N-7 ones [32]. Hence, the structures of the two isomers **1** and **1a** were assigned by comparing the ^1^H NMR spectra in DMSO-d_6_ in which the signals of the H-8, NH_2_, and the proton on the carbon atom linked to the imidazole nitrogen (N–CH) of the N-7 isomer, are downfield shifted compared with those of the N-9 isomer.

Finally, the alkylation site was unambiguously determinate by a chemical prove. In fact, hydrogenation of the 2-chloro-9-cyclopentyladenine (**1**), conducted in H_2_ atmosphere (30 psi) with Pd/C 10% in MeOH, gave the 2-unsubstituted derivative 9-cyclopentyladenine already described in the literature [32]. The reaction of **4** using the suitable cycloalkyl halides gave compounds **5** and **6**. In these cases, the reaction yields were so low that the N-7 isomers were not detected, so the structures of the two compounds were assigned on the base of the ^1^H NMR spectra.

**Scheme 2:**
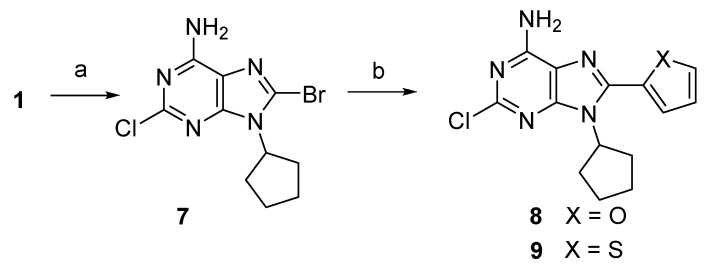
Reagents and conditions: a—NBS, MeCN and DMF, r.t., 48 h; b—tributylstannyl-aryl, PdCl_2_(PPh_3_)_2_, THF, 70 °C for 48 h.

For the synthesis of the 8-aryl derivatives **8** and **9**, a solution of *N*-bromosuccinimide (NBS) in MeCN was added dropwise to **1**, and solubilized in dry dimethylformamide (DMF). After 48 h, the reaction was complete and **7** was obtained with a 79% yield. Replacement of the **7** bromine atom with aromatic rings was performed via Stille reaction conditions, using the suitable tributylstannyl-aryl and bis(triphenylphosphine) palladium dichloride in tetrahydrofuran (THF) at 70 °C for 48 h. The derivatives **8** and **9** were obtained with 54% and 35% yields, respectively (Scheme 2).

**Scheme 3:**
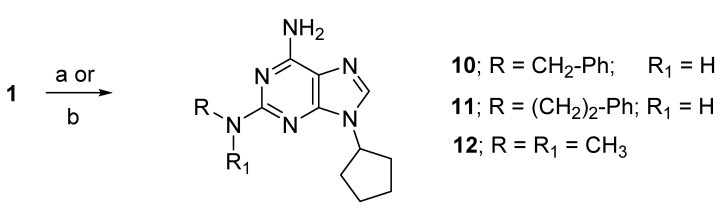
Reagents and conditions: a—benzylamine or 2-phenylethylamine, K_2_CO_3_, 130 °C, 24–72 h; b—dimethylamine, 120 °C, 12 h.

The 2-arylalkylamine derivatives **10** and **11** were synthesized by treating **1** with an excess of phenylethylamine or benzylamine, in the presence of K_2_CO_3_ in a sealed glass vial and heated at 130 °C for 24–72 h. Conversely, the 2-dimethylamino derivative **12** was obtained by treating **1** with dimethylamine in a sealed vial at 120 °C for 12 h (Scheme 3).

**Scheme 4:**
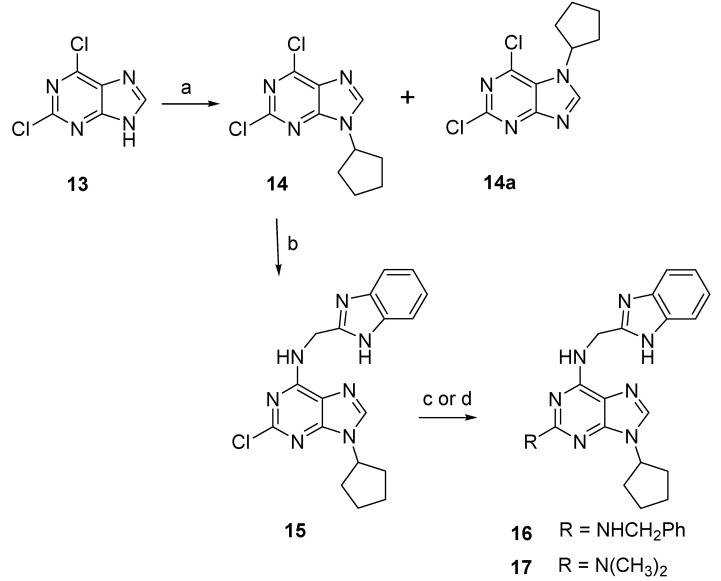
Reagents and conditions: a—cyclopentyl bromide, K_2_CO_3_, DMF, r.t., 48 h; b—2-aminomethyl-benzimidazole, Et_3_N, DMF, r.t., 30 h; c—benzylamine, K_2_CO_3_, 100 °C, 48 h; d—dimethylamine, 100 °C, 12 h.

The trisubstituted adenine derivatives **15**–**17** were obtained starting from the commercially available 2,6-dicloropurine (**13**), which was reacted with cyclopentyl bromide to give the intermediate **14** (Scheme 4). As in the case of compound **1**, the 9-cyclopentyl-2,6-dicloropurine **14** was obtained together with its N7 isomer **14a**. The structure of the two isomers was assigned, also in this case, on the base of the NMR spectra. The 2-chloro-9-cyclopentyl-*N*^6^-methyl-(2-benzimidazolyl)adenine (**15**) was obtained by replacing the chlorine atom in the 6-position of **14** with (2-benzimidazolyl)methylamine in dry DMF and Et_3_N, at r.t. for 36 h. Then, the 2-chlorine atom of **15** was replaced with benzylamine or dimethylamine at 100 °C for 12 h to give the desired **16** and **17**, respectively (Scheme 4).

**Scheme 5:**
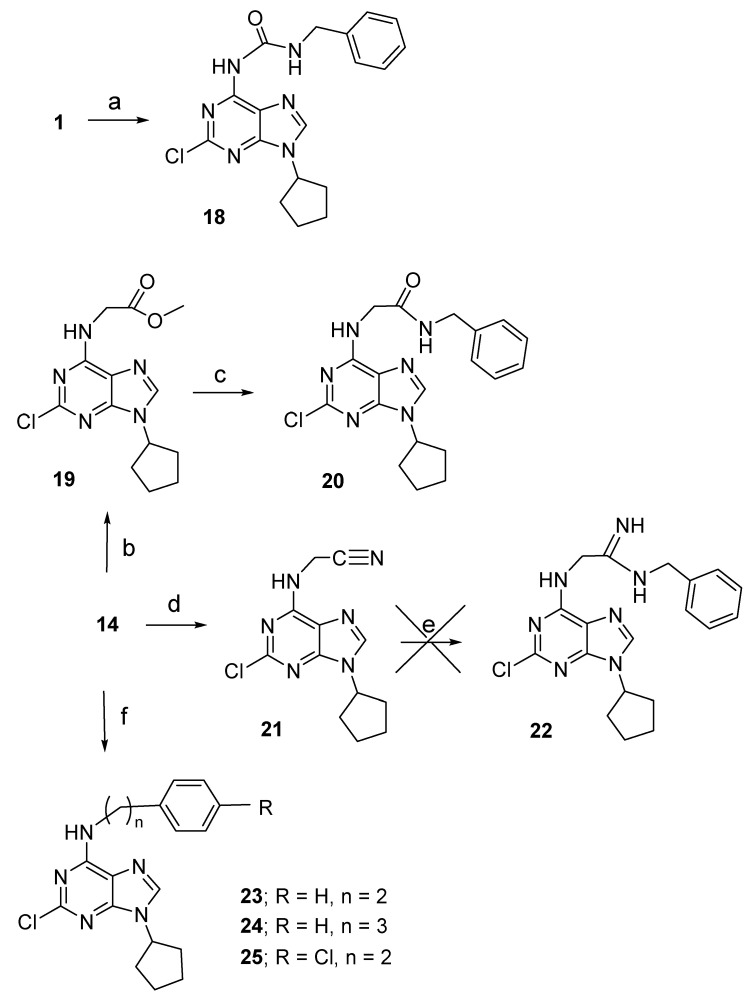
Reagents and conditions: a—benzyl isocyanate, Et_3_N, DMF, 100 °C, 24 h; b—methyl glycinate, Cs_2_CO_3_, CuI, THF, r.t., 6 h; c—benzylamine, 80 °C, 24 h; d—aminoacetonitrile, Et_3_N, THF, r.t., 18 h; e—benzylamine, r.t. to 100 °C, 24 h.; f—phenylethylamine or phenylpropylamine, K_2_CO_3_, MeCN, r.t. for 12 h; g—2 (p-chlorophenyl)ethylamine, Et_3_N, DMF, r.t., 24 h.

To obtain the methyl-(2-benzimidazolyl) simplified analogues **18**, **20**, **22**–**25**, different synthetic approaches were used. In particular, the urea analogue **18** was prepared by the reaction of **1** with benzyl isocyanate at 100 °C for 24 h (Scheme 5).

The *N*^6^-acetamido derivative **20** was, instead, obtained from the reaction of **14** with the methyl glycinate, in the presence of Cs_2_CO_3_ and CuI at r.t. for 6 h, to obtain the intermediate methyl acetate **19** (Scheme 5). Then, the latter was converted into the *N*^6^-benzylacetamido-2-chloro-9-cyclopentyladenine (**20**) via a reaction with benzylamine at 100 °C in the presence of Et_3_N.

An attempt to obtain the acetamido derivative **22** was performed, starting from the acetonitrile derivative **21** obtained by the reaction of **14** with aminoacetonitrile in anhydrous THF at 50 °C, with the catalysis of Et_3_N (Scheme 5). Unfortunately, the reaction of **21** with benzylamine led to a complex reaction mixture from where it was not possible to obtain the desired compound **22**. Other attempts to synthesize this derivative were unsuccessful.

Finally, the *N*^6^-arylalkyl derivatives **23**–**25** were obtained through the selective displacement of the **14** 6-chlorine atom with the suitable arylalkylamine in MeCN and the basic condition for K_2_CO_3_ at r.t. (Scheme 5).

### 2.2. CK1δ Inhibitory Activity Assay

The newly synthesized compounds **1**, **5**, **6**, **8**–**12**, **15–21**, and **23**–**25**, including some intermediates, were evaluated on truncated CK1δ using the luminescent kinase assay Kinase Glo^®^ KIT (Promega) at a fixed dose of 40 μM [11]. For compounds that showed values of protein residual activity lower than 50, the experiment was replicated at a fixed dose of 10 μM. The IC_50_ values were calculated for compounds with a CK1δ residual activity lower than 50%. The results are reported in Table 1 together with the data of the reference compound **1**. As reported above, the 2-chloro-9-cyclopentyladenine (**1**) displayed an IC_50_ = 5.20 μM and left an enzyme residual activity of 0.70% and 13% at 40 μM and 10 μM, respectively. The newly synthesized adenine derivatives showed values of CK1δ residual activity <50% at the dose of 40 μM with the exception of compounds **8**, **9**, and **11**, for which the activity at the fixed dose of 10 μM was not calculated and the IC_50_ values were estimated at >40 μM . On the other hand, at the dose of 10 μM, only compound **6** left an enzyme residual activity >50% and an estimated IC_50_ > 10 μM. Replacement of the **1** cyclopentyl ring with a cyclobutyl substituent led to the maintenance of inhibitory activity; in fact, **5** exhibited an IC_50_ = 5.25 μM, while the presence in the 9-position of a cycloalkyl ring was disadvantageous for CK1δ inhibitory activity (**6**; IC_50_ > 10 μM).

The introduction of a 2-furyl and a 2-thienyl group in the 8-position of the 2-chloro-9-cyclopentyladenine (**1**) was, instead, detrimental for the activity (**8** and **9**; IC_50_ > 40 μM). Substitution of the **1** 2-chlorine atom gave different results depending on the type of substituent. In this case, the presence of a benzylamino group was well tolerated (**10**: IC_50_ = 5.30 vs. **1**; IC_50_ = 5.25), while a longer phenethylamino substituent was detrimental for enzyme inhibitory activity (**11**; IC_50_ > 40 μM). A good result was obtained with the replacement of the chlorine atom with a dimethylamino group, which led to a 3-fold increase of inhibitory activity (**10**: IC_50_ = 1.75 μM). As expected, the further substitution of these derivatives with a methyl-(2-benzoimidazolyl) ring at the *N*^6^-position led to an increase of the activity that was more pronounced (about 3-fold) in compounds bearing a 2-chloro and a 2-dimethyilamino groups (**15**; IC_50_ = 1.53 μM and **17**; IC_50_ = 0.59 μM). Good results were also obtained when the *N*^6^-methyl-(2-benzylimidazolyl) substituent of the 2-chlorine derivative **15** was simplified. In particular, replacement of the *N*^6^-methyl-(2-benzylimidazole with an amido or ester group led to the maintenance of or a small decrease in the inhibitory activity, while the presence of the simpler cyanomethyl, phenethyl and phenylpropyl groups led to compounds with enhanced activity. In fact, compound **20**, bearing a benzylamidomethyl group at the *N*^6^-position, retained the activity of **15** with an IC_50_ = 1.83 μM, while the *N*^6^-acetomethylester **19** and the *N*^6^-benzylamido derivative **18** showed a slight decrease in the inhibitory activity with an IC_50_ = 2.55 μM and an IC_50_ = 3.37 μM, respectively.

Better results were obtained with compounds endowed with more simplified *N*^6^-substituents; in fact, **23** and **24**, bearing a phenethyl and a phenylpropyl group, exhibited IC_50_ values of 0.66 μM and 0.73 μM, respectively. It is worthwhile to note that the extreme simplification of the methyl-2-benzylimidazole with a cyanomethyl group gave the best result, leading to the most active compound of the series (**21**: IC_50_ = 0.36 μM).

### 2.3. Binding Assay at A_1_, A_2A_, and A_3_ ARs and Functional Studies at A_2B_ARs

Compounds **5**, **6**, **8**–**12**, **15**–**21**, and **23**–**25** were evaluated in binding studies at human recombinant A_1_, A_2A_, and A_3_ ARs, stably transfected in Chinese hamster ovary (CHO) cells. [^3^H]CCPA (2-chloro-*N*^6^-cyclopentylAdo), [^3^H]NECA (5’-N-ethylcarboxamidoAdo), and [^3^H]HEMADO (2-hexynyl-*N*^6^-methylAdo) were used as the, respective, radioligands [33,34]. All compounds were also tested at the A_2B_ARs by inhibition of NECA-stimulated adenylyl cyclase activity, using the GloSensor cAMP assay, but none of them were active at a concentration up to 30 μM [35]. The results, together with the data of the reference adenine derivative **1**, are reported in Table 2 as Ki values in μM (± standard errors). In the table, the IC_50_ values (μM) of the CK1δ inhibitory activity are also indicated. As already reported, **1** shows a moderate affinity for the A_2A_AR and a slight selectivity for the same subtype. The newly synthesized derivatives exhibited an affinity at the A_1_, A_2A_, and A_3_ ARs with Ki values ranging from low μM to low nM values and most of them showed a preference for the A_2A_ARs. Replacement of the cyclopentyl ring of **1** with a cyclobutyl group led to a derivative that maintained a comparable A_2A_AR affinity (**5**; KiA_2A_ = 0.558 μM versus **1**; KiA_2A_ = 0.465 μM). On the other hand, their ability to also inhibit the CK1δ enzyme was similar (**5**; IC_50_ = 5.25 μM versus **1**; IC_50_ = 5.20 μM). The affinity at the ARs of the 9-cyclohexyl derivative **6** has not been determined, since it was obtained with a very low yield and this resulted in it being poorly active as a CK1δ inhibitor. It is worthwhile to note that the derivatives endowed with the highest affinity at the A_2A_AR showed very low CK1δ inhibitory activity (**8**; KiA_2A_ = 0.007 μM, IC_50_ > 40 μM and **11**; KiA_2A_ = 0.012 μM, IC_50_ > 40 μM). Even the 2-chloro-9-cyclopentyl-8-(2-thienyl)adenine (**9**), which binds the A_1_, A_2A_, and A_3_ ARs with a Ki of 0.053 μM, 0.036 μM, and 0.017 μM, respectively, did not exhibit significant inhibitory activity (IC_50_ > 40 μM). On the other hand, the most potent enzyme inhibitor of the series was found to possess moderate affinity at ARs with a Ki in the μM range (**21**; KiA_1_ = 8.657 μM, KiA_2A_ = 3.478 μM, KiA_3_ = 5.957 μM, and IC_50_ = 0.36 μM). However, the disubstituted derivative **12** showed both good A_2A_AR affinity and enzyme inhibitory activity (**12**; KiA_2A_ = 0.123 μM, IC_50_ = 1.75 μM). It is worthwhile to note that the trisubstituted adenine derivative 9-cyclopentyl-2-dimethylamino-*N*^6^-methyl-(2-benzimidazolyl)adenine resulted in the compound being endowed with the best balance between A_2A_AR affinity and CK1δ inhibitory activity (**17**; KiA_2A_ = 0.076 μM, IC_50_ = 0.59 μM). As well, the trisubstituted derivatives **23** and **24** showed good receptor affinity, but in these cases, at the A_3_AR and A_1_AR, respectively, and CK1δ inhibition (**23**; KiA_3_ = 0.151 μM, IC_50_ = 0.66 μM and **24**; KiA_1_ = 0.692 μM, IC_50_ = 0.73 μM). We called the compounds endowed with receptor antagonist behavior combined with an enzyme inhibitory activity “dual anta-inhibitors”. Hence, **17** represents the first lead compound of the “dual anta-inhibitors” of the A_2A_AR and CK1δ enzyme ever reported.

### 2.4. Functional Activity at Human A_2A_AR

In order to confirm the antagonist activity of the dual anta-inhibitors **12** and **17**, they were evaluated in a functional assay in CHO cells stably expressing the hA_2A_AR. Firstly, they were tested alone, and any response in terms of cAMP stimulation was observed. Hence, their antagonist profile was evaluated by assessing their ability to counteract the NECA-induced increase in cAMP accumulation [34]. Both **12** and **17** were able to inhibit the effect of NECA on cAMP production, thus resulting in A_2A_AR antagonists. The calculated IC_50_ (μM) values were 0.418 ± 0.098 for **12** and 0.069 ± 0.018 for **17**. Hence, the functional study confirmed **17** as a more potent A_2A_AR antagonist than **12**.

### 2.5. Computational Studies

To characterize, at the molecular level, the protein–ligand interactions involving the compounds of our series, a computational study was executed. Indeed, computer-based methods are largely exploited, both in academic and industrial scenarios, to speed up the discovery process of new efficacious molecules and to understand the ligand–target contact patterns [36]. This is crucial to improve the understanding of QSAR analysis and guide further optimization steps in drug design [37].

In the present study, our approach started with the proper preparation procedure for both ligands (the 18 molecules of our series, reported in Table 1 and Table 2) and the proteins (CK1δ and the three AR isoforms A_1_AR, A_2A_AR, and A_3_AR), to make them suitable for computational handling. Following the cross-docking study reported in a recent article by Pavan and Menin et al. [38], the crystal structure chosen for CK1δ was the one with PDB code 4TN6 (method: X-ray diffraction, resolution: 2.41 Å), while the AR complexes were chosen by using the crystallographic resolution criteria among the ones complexed with a non-covalent small organic molecule available in the Protein Data Bank [39]. Finally, the structures chosen were, respectively, 7LD4 for A_1_AR (method: cryo-EM, resolution 3.3 Å) [40], 5K2C for A_2A_AR (method: X-ray diffraction, resolution: 1.9 Å) [41], while A_3_AR, as it was not presenting any available experimental structure, was created by homology modeling based on the already cited A_1_AR complex [42]. The specific passages executed for the preparation of such chemical and biological entities are reported in Section 3.

Then, the adenine molecules were docked in the orthosteric binding site of all of the four receptors listed using the PLANTS program (which is developed and distributed by the University of Tübingen and is based on an Ant Colony Optimization algorithm) [43], using PLANTS_CHEMPLP_ as the scoring function. The binding site for the docking was chosen by taking the coordinates of the center of the mass of the crystallographic ligand as a reference and creating a sphere of 15 Å radius from it. For each molecule in the database, 25 poses were generated, which were then filtered to exclude all the conformations presenting steric clashes or unfavorable electrostatic interaction with the protein. The remaining poses were then prioritized following the available structure-activity information for each receptor. Indeed, several studies have already been performed in the past by several academic and industrial groups about CK1δ and ARs, including us, and the most important features to guarantee stable binding with the receptors have been elucidated [44,45,46,47,48,49,50,51,52].

#### 2.5.1. CK1δ Inhibition

**Figure 4:**
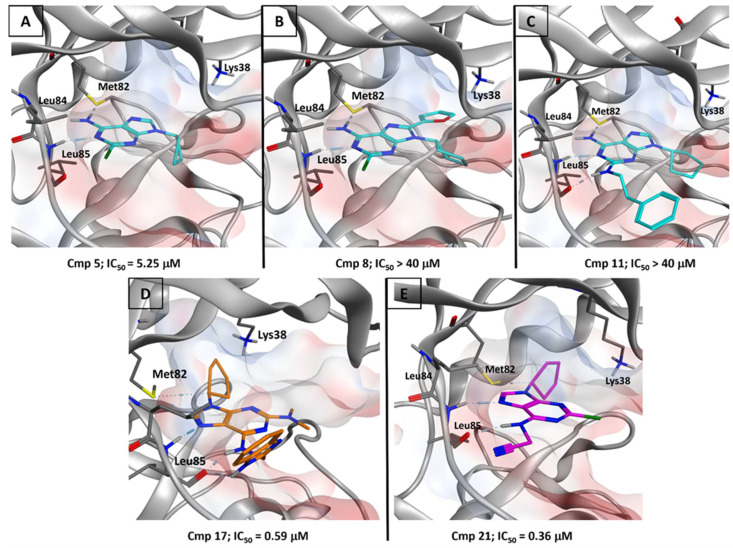
Depiction of the three-dimensional structure of the selected poses for some of the representative compounds tested for CK1δ inhibition. Panels (**A**–**E**) represent, respectively, compounds **5**, **8**, **11**, **17**, and **21**. For each image, the most important CK1δ residues are labeled, and the electrostatic surface of the pocket is mapped and illustrated. The ligands are colored based on the “subseries” in which the molecules have been divided in Table 1. Compounds **5**, **8**, and **11** are colored in cyan, molecule **17** is colored orange, while ligand **21** is depicted in purple. The hydrogen bonds are indicated with dashed lines. All of the images were created and rendered with MOE.

As can be seen, the pocket of CK1δ is characterized by the hinge region, which is formed by the residues from Met82 to Leu85 and represents an anchor point for ATP and the vast majority of orthosteric inhibitors. Then, a small hydrophobic pocket (Figure 4, between Met82 and Lys38), also referred to as a “selectivity pocket”, is present in the posterior part of the binding site. The ATP-binding pocket is denoted by an overall narrow shape that leads, to the right side, to the residue Lys38. Adenine inhibitors typically interact with CK1δ by a fundamental hydrogen bond with the hinge region, as can be seen in all of the poses selected for the molecules considered in this study (Figure 4). Although this is a fundamental anchoring interaction, it is not sufficient to guarantee that the ligand exhibits high potency toward the target. Indeed, this interaction is also present for the low-potency compounds tested (as can be seen in Figure 4B,C), leading us to look at other factors to discriminate the quality of the binding.

First, the QSAR obtained from the in vitro test makes us understand the important role of the conformational freedom of the ligand for its binding efficiency. Indeed, as we can see, passing from the cyclopentane or cyclobutane moiety in the 9-position for compounds **1** and **5**, to the cyclohexane of compound **6**, causes a direct, small, reduction in potency. The same is true passing from the dimethylamino to the benzylamino and then to the phenylethylamino groups in compounds **12**, **10**, and **11**, respectively. Indeed, in both the cited cases, the chemical groups that are changing are oriented outwards in relation to the binding pocket, not interacting directly within it (except for the amine group in the chain of **10** and **11**, which can be involved in hydrogen bonds with the hinge region). What can be important to consider in this scenario is how the augmented conformational freedom of the molecule impacts its dynamic interaction events with the target. Indeed, even if, from the static view offered by molecular docking, this could seem to be a minor effect, it can become very important if we examine the protein–ligand interaction process. Looking at Figure 4, we can see that the ligands numbered from **15** to **25**, having a substituent in the R_4_-position, display a binding mode that is “reversed” compared to the first group of compounds, always making contact with the hinge region, but using the 7-nitrogen atom and the 6-amino group instead of the 1-nitrogen atom of the adenine core. This change in the orientation of the molecule allows them to direct the cyclopentyl group towards the selectivity pocket, exploiting the space inside the cavity better. Moreover, the best activity profile is shown by the compounds with the smallest group in the R_4_-position, probably due to the simplified dynamic recognition path regarding the receptor. Indeed, all of the ligands numbered from **18** to **25** show potency in the high-nM to the low-µM range, allowing us to assess that one of the discriminants between them could be the amount of different interactive modes with CK1δ. When approaching the receptor, a ligand orients its groups in the proper conformation to proficiently bind to the target [53]. If these conformations are multiple and if there are more groups in the molecule able to establish the same kind of contact with the pocket (e.g., the carbonyl group of compounds **18**, **19**, and **20** can interact with the hinge region), it can both take more time and be less probable for the ligand to correctly assume the most proficient conformation. This is reflected by the slight reduction in potency for the “bigger” ligands in the **18** to **20** and **23** to **25** ranges of our series.

#### 2.5.2. Adenosine Receptor Inhibition

**Figure 5:**
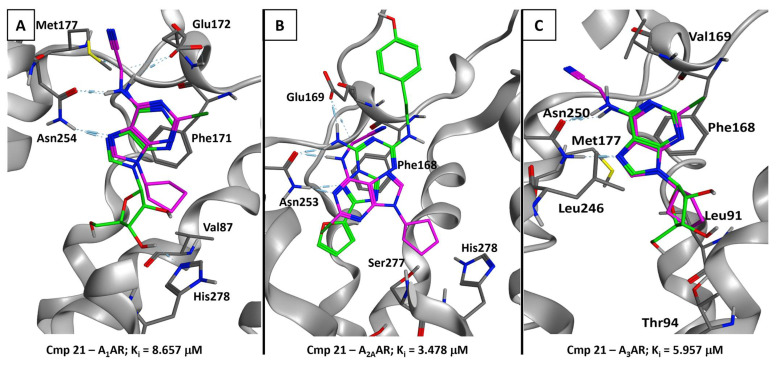
Representation of the three binding modes of compound **21** in complex with, respectively, the A_1_AR (panel (**A**)), A_2A_AR (panel (**B**)), and A_3_AR (panel (**C**)). In each image, the reference compound is colored light green (Ado for A_1_AR and A_3_AR; ZMA for A_2A_AR), while compound **21** is purple. The main residues surrounding the ligands in the binding site are labeled, and the hydrogen bonds are represented with dashed lines. All of the images were created and rendered with MOE.

Looking at the poses obtained for the molecule of our series on the A_1_AR, A_2A_AR, and A_3_AR, we can see that the recognition pattern typical of the Ado ligands is kept for all the different targets. This is in line with the experimental data, which show that almost all of the compounds exhibit an activity between the nM and the low-µM range in all of the receptor isoforms. To obtain a visual representation of the interaction patterns, we displayed, in Figure 5, the selected docking poses of the most potent CK1δ compound, the one numbered **21**, in the orthosteric binding site of A_1_AR (Figure 5A), A_2A_AR (Figure 5B), and A_3_AR (Figure 5C). In each of the panels of Figure 5, the binding mode of compound **21** is compared with the conformation of the crystallographic ligand of the specific receptor. For the A_1_AR and A_3_AR models, the crystal pose of Ado is also reported, while for A_2A_AR, the conformation of the antagonist ZM 241385 (ZMA) is depicted [54].

**Figure 6:**
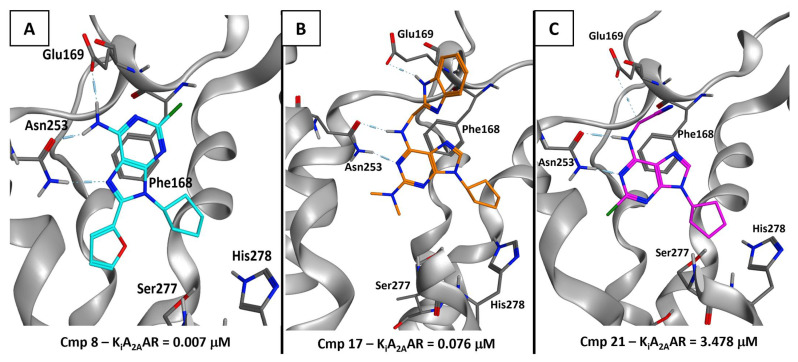
Depiction of the three poses selected for compounds **8** (colored in cyan, panel (**A**)), **17** (in orange, panel (**B**)), and **21** (in purple, panel (**C**)) in complex with A_2A_AR. In each image, the main residues surrounding the ligands in the binding site are labeled, and the hydrogen bonds are represented with dashed lines. All of the images were created and rendered with MOE.

As can be seen, the important dual hydrogen bond with the asparagine residue in TM6 is conserved in all of the isoforms (Asn254 for A_1_AR, Asn253 for A_2A_AR, Asn250 for A_3_AR), together with the stacking between the adenine rings and the sidechain of the nearby phenylalanine (Phe171 in A_1_AR, Phe168 in both A_2A_AR and A_3_AR). Moreover, even if the salt bridge with the glutamate residue in the outer part of the pocket (Glu172 in A_1_AR, Glu169 in A_2A_AR) cannot be established because of the substitution in R_4_, a similar interaction can be exploited, seen via the dipole present on the CH_2_ carbon linking the cyano group with the adenine 6-amine of compound **21**. Indeed, the electro-withdrawing effect of the nitrile group can be addressed as the main factor responsible for such an interaction. We also represent, in Figure 6, the comparison between the binding modes of compounds **8**, **17**, and **21** within the A_2A_AR orthosteric site, given the fact that compound **8** is the best inhibitor of this receptor isoform (KiA_2A_AR = 0.007 μM) and molecule **17** has shown the best balance between A_2A_R and CK1δ inhibition (KiA_2A_AR = 0.076 μM, IC_50_CK1δ = 0.59 μM). For this specific balance, we will refer to compound **17** as the main “dual anta-inhibitor” of the A_2A_AR and CK1δ enzyme.

**Figure 7:**
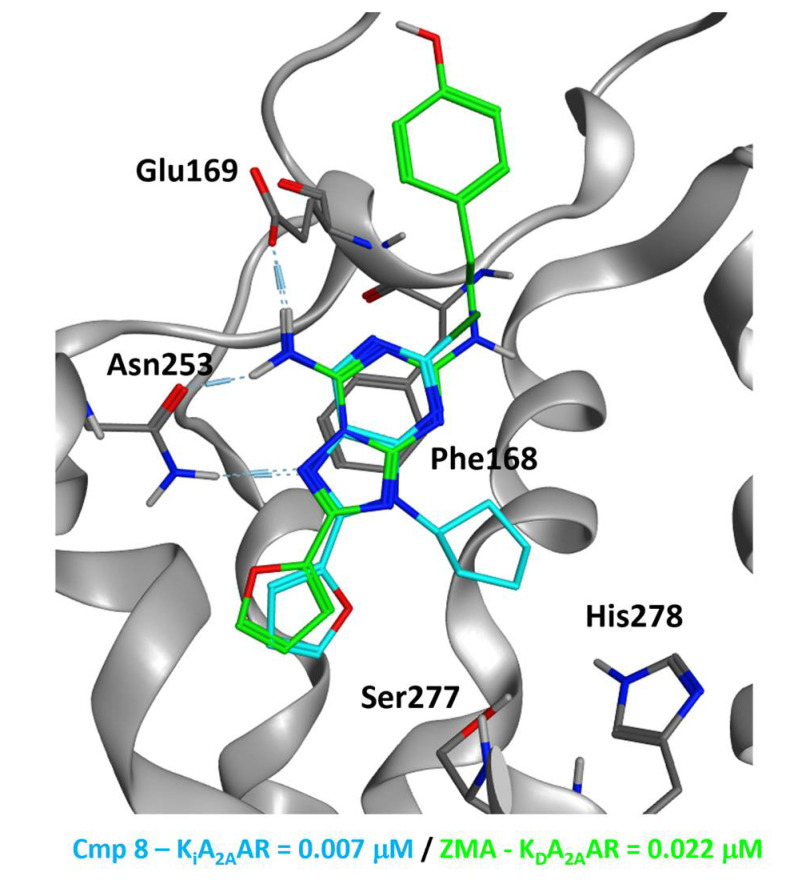
Comparison between the selected pose of compound 8 (colored in cyan, K_i_A_2A_AR = 7 nM) and the crystallographic conformation of ZM 241385 (ZMA, taken from the crystal with PDB code 5K2C, colored in light green, K_D_A_2A_AR = 22 nM). The main residues surrounding the two molecules in the pocket are labeled, and the hydrogen bonds are represented with dashed lines. All of the images were created and rendered with MOE.

The hydrogen bond with Glu169 seems to be related, to some extent, to the change in potency. Indeed, compound **8** can fully recruit it with its 6-amine group, while the “dual anta-inhibitor” **17** can also establish a hydrogen bond with it despite causing a little distortion in the glutamate sidechain (which was modeled with a very small amount of energy minimization of the group). Compound **21**, on the other hand, is just able to use the electron-poor CH_2_ carbon atom linked to the 6-amine group to contact Glu169. Moreover, compound **8** can proficiently occupy the inner part of the GPCR pocket, putting two groups (the cyclopentyl and the 2-furyl) deep into the binding site, while the other two molecules can do this with just the cyclopentyl moiety. To witness the effective goodness of the binding mode of compound **8**, we compared it with the crystallographic conformation of ZMA, a well-known A_2A_AR inhibitor, which also shares the 2-furyl moiety in the 8-position. The superposition of the pose selected for compound **8** and the cited ZMA crystal pose is represented in Figure 7.

####  2.5.3. Supervised Molecular Dynamics (SuMD) Simulations

As we mentioned previously, some of the most relevant molecular aspects discriminating the on-target efficacy of the compounds of our series could be related to their dynamic behavior, and most importantly, to the steps taken by these entities in approaching and binding to the biological target. Among the various possible approaches, one that has been extensively used for the evaluation of these molecular events is Supervised Molecular Dynamics (SuMD). SuMD is an enhanced-sampling method able to simulate the protein–ligand interaction pathway on a reduced timescale, exploiting a tabu-like supervision algorithm on the distance between the centers of mass of the ligand and the binding site, respectively [55]. Specifically, such a method has been successfully used on different targets in different scenarios [48,56,57,58]. To examine the dynamic aspects of the binding process between the most relevant ligands in our library, we executed SuMD simulations on both the CK1δ and A_2A_AR proteins, taking as a reference the most potent molecule against CK1δ (compound **21**) and the “dual anta-inhibitor” **17**. The videos of the trajectories obtained, together with the per-residue time-dependent interaction plot for each of them, are reported in the Supplementary Materials (“Video_S1.mp4”, “Video_S2.mp4”,“Video_S3.mp4”, and “Video_S4.mp4”).

**Figure 8:**
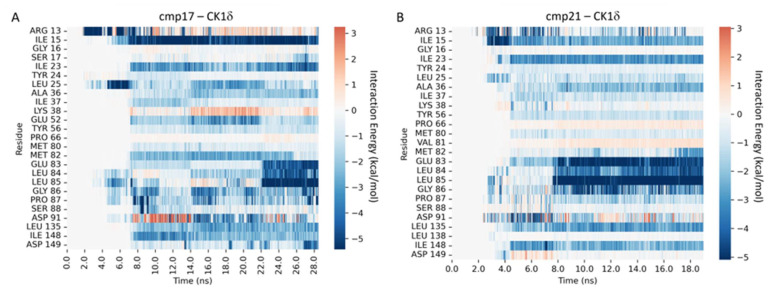
Per-residue decomposition of the protein–ligand interaction energy descriptive of the recognition process between protein kinase CK1δ and either the “dual anta-inhibitor” (compound **17**) (**A**) or compound **21** (**B**) sampled with Supervised Molecular Dynamics (SuMD) simulations.

**Figure 9:**
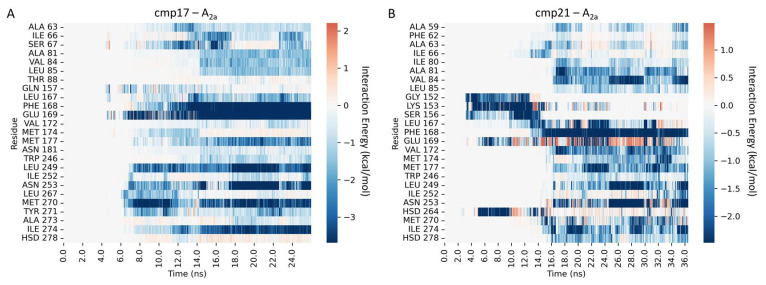
Per-residue decomposition of the protein–ligand interaction energy descriptive of the recognition process between A_2A_AR and either the “dual anta-inhibitor” (compound **17**) (**A**) or compound **21** (**B**) sampled with Supervised Molecular Dynamics (SuMD) simulations.

Concerning compound **21**, about 18 ns of SuMD simulation were sufficient to sample a putative recognition pathway towards CK1δ, while 36 ns were necessary to investigate the binding pathway towards the A_2A_AR. In both cases, the final state of the SuMD simulations converged both from a geometric and an interactive point of view with the predicted-docking binding mode. Regarding CK1δ inhibition, compound **21** can enter the kinase active site quite rapidly, thanks to the absence of any hindrance carried out by bulky substituents. As can be seen in “Video_S2.mp4” (Supplementary Materials), the ligand establishes its first contact with the residues located in the β-barrel domain, such as Arg13 and Ile15, around the 3 ns time mark. Particularly, the flexible, positively charged sidechain of Arg13 plays an important part in ensuring the right orientation of the ligand within the binding site, by recruiting the partially negative nitrile group and allowing the hydrophobic core of the molecule to enter the catalytic site head-first, resulting in the formation of a bivalent hydrogen bond with Leu85, a series of hydrophobic contacts of the cyclopentyl moiety within the selectivity pocket, and other favorable short-range interactions with the hinge region, as highlighted in our interaction energy heatmaps (Figure 8B). As previously mentioned, all these interactions are considered pivotal for effective CK1δ inhibition, thus justifying the good activity of this compound towards this target. Concerning the A_2A_AR recognition pathway, compound **21** establishes its first contacts with the residues located in the extracellular loop 2, such as Gly152, Lys153, and Ser156 around the 4 ns time mark, as depicted in “Video_S4.mp4” (Supplementary Materials). After spending a 12 ns resting period in a meta-stable binding site defined by the aforementioned residues, Glu169, and His264, the ligand revolves the cyclopentyl moiety head-first towards the core of the orthosteric site, establishing some transient contacts with residues such as Ala63, Ile66, and Leu167 before burying itself within the binding site and making stable interactions with residues such as Phe168, Asn253, Val84, Met177, Leu249, and Ile274 (Figure 9B), resulting in a binding pose that is fairly superimposable onto the predicted-docking one. Despite its ability to recognize the orthosteric site of A_2A_AR with the same binding determinants that are required for antagonism, the simulation shows how compound **21** spends a fair amount of time in the vestibular region of the binding site. A similar behavior was already noticed in a previous work from our laboratory [59], where we performed SuMD simulations of Ado (the endogenous agonist) on the inactive form of the receptor (the same utilized in this work, the one responsible for the recognition of antagonists) that ended with the ligand being stuck in the same meta-binding site explored by compound **21**, without ever reaching the orthosteric binding site. The ligand sequestration by this meta-stable binding site could be a first hint at explaining the different activity profile of compound **21** compared to the one of compound **17**.

The “dual anta-inhibitor” (compound **17**) shows a comparable binding pathway towards protein kinase CK1δ to the one collected for compound **21** (“Video_S1.mp4”, Supplementary Materials), although with different timings. In this case, also, the ligand approaches the β-barrel domain first, once again establishing its first contact with the enzyme through residues such as Arg13 and Ile15. Once again, Arg13 plays the “electrostatic recruiter” role and maneuvers the ligand towards the catalytic site, ensuring the right orientation upon entrance. Differently from compound **21**, which interacted through its nitrile tail with Arg13, the indole moiety of the “dual anta-inhibitor” (compound **17**) anchors the ligand to a small hydrophobic pocket located within the β-barrel region, opposing resistance to the dragging power of the “electrostatic recruiter”, as highlighted by the increased time required to place the “pseudo-adenine” core of the molecule nearby the hinge region. This delayed entrance within the binding site, in addition to the hindrance portrayed by the indole moiety, also causes a delay in the establishment of a strong and stable interaction with the residues populating the hinge region, as highlighted by our interaction energy heatmap (Figure 8A). These differences in the recognition pathway could be a possible explanation for the different enzymatic inhibition activity of the two compounds, regardless of a superimposable binding mode. The role of the indole substituent in altering the kinetics of the binding process is better elucidated by the analysis of the recognition pathway of compound **17** towards the A_2A_AR (“Video_S3.mp4”, Supplementary Materials). Contrary to what was observed for CK1δ recognition, where the smaller, less hindered ligand (compound **21**), had a faster binding process, in the case of the A_2A_AR, compound **17** is much quicker in reaching the final binding pose within the orthosteric site (only 24 ns of simulation time, compared to the 38 ns required for compound **21**). This is due to less time spent, by the ligand, within the meta-stable binding site located between the extracellular loops 2 and 3. Indeed, the “dual anta-inhibitor”(compound **17**) approaches the orthosteric site from the opposite side, establishing the first contact with the region comprised between the extracellular loops 1 and 3. Intriguingly, a similar behavior has recently been reported for the “non-ribose” partial agonist LUF5833 [48]. In the work of Bolcato et al., this binding pattern is exclusive to the “non-ribose” partial agonist LUF5833, in contrast with classic “ribose” agonists, which explore the same meta-binding site as compound **21**.

These observations concerning the binding pathways could complement previous considerations of the sequestration of agonists by this meta-stable site well, providing a plausible explanation for the different receptor inhibition activity of these compounds despite a superimposable binding mode. Without any obstacle to its entrance within the core of the binding site, compound **21** rapidly reaches a similar binding pose to the predicted-docking one, although slightly shifted towards the outer portion of the binding site. After a rearrangement of the indole moiety through the sidechain of Glu169, the ligand is finally locked in the classic binding pose for A_2A_AR antagonists, with the double hydrogen bond with Asn253, and the π-stacking with Phe168, as the main interaction determinants, coupled with other ancillary interactions with residues such as Met177, Leu249, Met270, and Ile274, as highlighted in our interaction energy heatmaps (Figure 9A). As anticipated when discussing the difference in CK1δ recognition, the different recognition pathways and binding kinetics showcased by SuMD simulations could provide a plausible mechanistic explanation of the different activity profiles of these two compounds, especially in the context of A_2A_AR antagonism.

## 3. Materials and Methods

### 3.1. Chemical Synthesis

#### General Methods

Melting points were determined with a Büchi apparatus and are uncorrected. ^1^H NMR spectra were obtained with a Bruker Ascend 500 MHz spectrometer (Bruker Italia S.r.l., Milano, Italy); δvalues are in ppm, J values are in Hz. All exchangeable protons were confirmed by the addition of D_2_O. Mass spectra were recorded on an HP 1100-MSD series instrument. Thin-layer chromatography (TLC) was carried out on pre-coated TLC plates with silica gel 60 F_254_ (Merk Life Science S.r.l., Milan, Italy). For column chromatography, silica gel 60 (Merck) or the Isolera Biotage four instrument was used. Elemental analyses were determined on a Fisons Instruments Model EA 1108 CHNS-O model analyzer and are within 0.4% of theoretical values. Purity of the compounds is ≥99% according to elemental analysis data.

The general procedure for the synthesis of 2-chloro-9-cyclopentyl-9*H*-purin-6-amine (**1**), 2-chloro-7-cyclopentyl-7*H*-purin-6-amine (**1a**), 2-chloro-9-cyclobutyl-9*H*-purin-6-amine (**5**), and 2-chloro-9-cyclohexyl-9*H*-purin-6-amine (**6**): **4** (100 mg; 0.59 mmol) was, in turn, reacted with 0.72 mmol of bromo cyclopentane (74 µL) or bromo cyclobutane (68 µL) or cyclohexyl iodide (92 µL) and K_2_CO_3_ (165 mg; 1.20 mmol) in dry DMF (3 ml) at r.t. under a nitrogen atmosphere and under stirring for 5 days. The reaction mixtures were left under stirring for 5, 4, and 2 days, respectively, then the volatiles were evaporated to dryness and the residues purified through a flash column silica gel, eluting with DCM-cHex-MeOH (85:10:5) to afford **1** (88 mg, 0.37 mmol; yield 63%) and **1a** (9 mg, 0.04 mmol; yield 6%), with DCM-cHex-MeOH (78:20:2) to obtain **5** (10 mg, 0.05 mmol; yield 7%), and with DCM-cHex-MeOH (70:28:2) to give **6** (7 mg, 0.03 mmol; yield 5%) as a white powder after crystallization from isopropanol.

Compound **1**: m.p. 121–122 °C. ^1^H-NMR (DMSO-d_6_) δ: 1.58 (m, 2H, CH_2_), 1.87 (m, 2H, CH_2_), 2.00 (m, 2H, CH_2_), 2.11 (m, 2H, CH_2_), 4.77 (m, 1H, CH); 7.72 (s, 2H, NH_2_); 8.24 (s, 1H, H-8). ESI-MS, positive mode *m*/*z*: 238.0 ([M+H]^+^); 260.0 ([M+Na]^+^). Elemental analysis calcd for C_10_H_12_ClN_5_: C, 50.53; H, 5.09; N, 29.46; found C, 50.45; H, 5.12; N, 29.39.

Compound **1a**: m.p. 150–152 °C. ^1^H-NMR (DMSO-d_6_) δ: 1.87 (m, 2H, CH_2_), 1.87 (m, 2H, CH_2_), 1.95 (m, 2H, CH_2_), 2.16 (m, 2H, CH_2_), 4.81 (m, 1H, CH), 8.28 (s, 2H, NH_2_); 8.85 (s, 1H, H-8). ESI-MS, positive mode *m*/*z*: 238.0 ([M+H]^+^); 260.0 ([M+Na]^+^). Elemental analysis calcd for C_10_H_12_ClN_5_: C, 50.53; H, 5.09; N, 29.46; found C, 50.49; H, 5.18; N, 29.41.

Compound **5**: m.p. 117–119 °C. ^1^H-NMR (DMSO-d6) δ: 1.77 (m, 2H, CH_2_), 3.41 (m, 4H, CH_2_), 4.95 (m, 1H, N-CH), 7.45 (bs, 2H, NH_2_), 8.70 (s, 1H, H-8). ESI-MS, positive mode *m*/*z*: 224.2 ([M+H]^+^); 246.2 ([M+Na]^+^). Elemental analysis calcd for C_9_H_10_ClN_5_: C, 48.33; H, 4.51; N, 31.31; found C, 48.57; H, 4.24; N, 31.15.

Compound **6**: m.p. 125–126 °C. ^1^H-NMR (DMSO-d6) δ: 1.24 (m, 2H, CH_2_), 1.44 (m, 2H, CH_2_), 1.70 (m, 2H, CH_2_), 1.83 (m, 2H, CH_2_), 1.99 (m, 2H, CH_2_), 4.27 (m, 1H, N-CH), 7.71 (bs, 2H, NH_2_), 8.25 (s, 1H, H-8). ESI-MS, positive mode *m*/*z*: 258.2 ([M+H]^+^); 280.4 ([M+Na]^+^). Elemental analysis calcd for C_11_H_14_ClN_5_: C, 52.49; H, 5.61; N, 27.82; found C, 52.40; H, 5.71; N, 27.70.

8-Bromo-2-chloro-9-cyclopentyl-9*H*-purin-6-amine (**7**): Compound **1** (500 mg; 2.08 mmol) was dissolved in 9 mL of MeCN and a solution of NBS (555 mg; 3.12 mmol) in 5 mL of MeCN was added dropwise over the course of 30 min, under a nitrogen atmosphere. The mixture was stirred at r.t. for 48 h, then the volatiles were evaporated. H_2_O was added and the mixture was extracted with EtOAc (20 mL X 3). The organic layers were dried over Na_2_SO_4_, then filtered, evaporated, and purified by flash column chromatography, eluting with cHex-EtOAc (80:20). Compound **7** was obtained as a white solid (519 mg, 1.64 mmol; yield 79%). M.p.: 155–156 °C. ^1^H-NMR (DMSO-*d_6_*) δ: 1.60 (m,2H, CH_2_), 1.92 (m, 2H, CH_2_), 2.01 (d, 2H, CH_2_), 2.20 (m, 2H, CH_2_), 4.86 (m, 1H, CH), 7.86 (s, 2H, NH_2_). ESI-MS, positive mode *m*/*z*: 315.9 ([M+H]^+^); 338.9 ([M+Na]^+^). Elemental analysis calcd for C_10_H_11_BrClN_5_: C, 37.94; H, 3.50; N, 22.12; found C, 37.88; H, 3.45; N, 22.30.

The general procedure for the synthesis of 2-chloro-9-cyclopentyl-8-(furan-2-yl)-9*H*-purin-6-amine (**8**) and 2-chloro-9-cyclopentyl-8-(thiophen-2-yl)-9*H*-purin-6-amine (**9**): Compound **7** (100 mg; 0.31 mmol) was solubilized in THF (5 mL) and 0.62 mmol of 2-tributylstannylfurane (228 mg) or 2-tributylstannylthiophene (231 mg) and PdCl_2_(PPh_3_)_2_ (10 mg, 0.02 mmol) were added. The mixture was left in a sealed glass vial at 70 °C for 48 h. The volatiles were evaporated and the residues purified by flash column chromatography using Isolera Biotage, eluting with C-Hex-EtOAc (80:20) to obtain **8** (51 mg, 0.16 mmol; yield 54%) and **9** (34 mg, 0.11 mmol; yield 35%) as white solids.

Compound **8**: m.p.: 152–153 °C. ^1^H-NMR (DMSO-*d_6_*) δ: 1.67 (m, 2H, CH_2_), 1.98 (m, 2H, CH_2_), 2.09 (m, 2H, CH_2_), 2.27 (m, 2H, CH_2_), 5.11 (m, 1H, CH), 6.76 (t, *J* = 7.2 Hz, 1H, H-furyl), 7.11 (d, 1H, H-furyl), 7.83 (brs, 2H, NH_2_), 7.99 (d, *J* = 7.2 Hz, 1H, H-furyl). ESI-MS, positive mode *m*/*z*: 304.0 ([M+H]^+^); 325.0 ([M+Na]^+^). Elemental analysis calcd for C_14_H_14_ClN_5_O: C, 55.36; H, 4.65; N, 23.06; found C, 55.25; H, 4.55; N, 23.06.

Compound **9**: m.p.: 158–159 °C. ^1^H-NMR (DMSO-*d_6_*) δ:1.65 (d, 2H, CH_2_), 1.99 (d, 2H, CH_2_), 2.08 (d, 2H, CH_2_), 2.32 (d, 2H, CH_2_), 4.99 (m, 1H, CH), 7.28(t, *J* = 7.6 Hz, 1H, CH), 7.58 (d, *J* = 7.6 Hz, 1H, CH), 7.78 (brs, 2H, NH_2_), 7.85 (d, *J* = 7.5 Hz, 1H, CH). ESI-MS, positive mode *m*/*z*: 319.9 ([M+H]^+^). Elemental analysis calcd for C_14_H_14_ClN_5_S: C, 52.58; H, 4.41; N, 21.90; found C, 52.50; H, 4.48; N, 21.90.

*N*^2^-benzyl-9-cyclopentyl-9*H*-purine-2,6-diamine (**10**): In a glass sealed vial, compound **1** (100 mg; 0.42 mmol) was solubilized in dry DMF (5 mL). Benzylamine (92 µL; 0.84 mmol) and K_2_CO_3_ (348 mg; 2.52 mmol) were added to the solution and the mixture was left at 130 °C for 24 h. After the evaporation of the volatiles, H_2_O was added, and the mixture was extracted with EtOAc (20 mL X 3). The organic layers were dried over anhydrous Na_2_SO_4_, filtered, and the residue was purified by preparative TLC, eluting with DCM-MeOH (98:2). Compound **10** (7 mg, 0.03 mmol; yield 6%) was obtained as a white solid. M.p.: 165–166 °C. ^1^H-NMR (DMSO-*d_6_*) δ:1.63 (m, 2H, CH_2_), 1.82 (m, 2H, CH_2_), 1.93 (m, 2H, CH_2_), 2.01 (m, 2H, CH_2_), 4.45 (d, *J* = 6.1 Hz, 2H, CH_2_-N), 4.62 (m, 1H, CH), 6.61 (s, 2H, NH_2_), 6.76 (t, *J* = 7.2 Hz, 1H, NH), 7.26 (m, 5H, Ph), 7.73 (s, 1H, H-8). ESI-MS, positive mode *m*/*z*: 309.0 ([M+H]^+^); 331.0 ([M+Na]^+^). Elemental analysis calcd for C_17_H_20_N_6_: C, 66.21; H, 6.54; N, 27.25; found C, 66.10; H, 6.62; N, 27.24.

9-Cyclopentyl-*N*^2^-phenethyl-9*H*-purine-2,6-diamine (**11**): In a glass sealed vial, compound **1** (150 mg; 0.63 mmol) was suspended in 2-phenylethylamine (3 mL) and K_2_CO_3_ (261 mg; 1.89 mmol) was added. The mixture was left at 130 °C for 72 h, then H_2_O was added and the mixture was extracted with EtOAc (20 mL X 3). The organic layers were dried over anhydrous Na_2_SO_4_, then filtered and evaporated and the residue was purified by flash column chromatography, eluting with cHex-DCM-MeOH (70:28:2). Compound **11** (12 mg, 0.04 mmol; yield 6%) was obtained as a white powder. M.p.: 172–173 °C. ^1^H-NMR (DMSO-*d_6_*)δ: 1.66 (m, 2H, CH_2_), 1.87 (m, 2H, CH_2_), 2.01 (m, 2H, CH_2_), 2.10 (m, 2H, CH_2_), 2.84 (d, *J* = 7.0 Hz, 2H, CH_2_-Ph), 3.45 (d, *J* = 7.0 Hz, 2H, CH_2_-N), 4.66 (m, 1H, CH), 6.24 (t, *J* = 7.2 Hz, 1H, NH), 6.62 (brs, 2H, NH_2_), 7.26 (m, 5H, Ph) 7.75 (s, 1H, H-8). ESI-MS, positive mode *m*/*z*: 323.0 ([M+H]^+^); 345.0 ([M+Na]^+^). Elemental analysis calcd for C_18_H_22_N_6_: C, 67.06; H, 6.88; N, 26.07; found C, 67.12; H, 6.80; N, 26.05.

9-Cyclopentyl-*N*^2^,*N*^2^-dimethyl-9*H*-purine-2,6-diamine (**12**): To an excess of liquid dimethylamine, cooled with liquid nitrogen in a hermetically sealed vial, **1** (50 mg, 0.21 mmol) was added and the mixture was heated to 100 °C for 12 h. Then, the volatiles were evaporated and the mixture was purified by flash column chromatography, eluting with DCM-MeOH (95:5). Compound **12** was obtained as a white powder: (22 mg; 0.09 mmol; yield) 45%. M.p.: 185–187 °C. ^1^H-NMR (DMSO-*d_6_*) δ: 1.67 (m, 2H, CH_2_), 1.78 (m, 2H, CH_2_), 1.97 (m, 2H, CH_2_), 2.10 (m, 2H, CH_2_), 2.99 (s, 6H, 2xCH_3_), 4.62 (m, 1H, CH), 6.62 (brs, 2H, NH_2_), 7.73 (s, 1H, H-8). ESI-MS, positive mode *m*/*z*: 247.1 ([M+H]^+^); 369.2 ([M+Na]^+^). Elemental analysis calcd for C_12_H_18_N_6_: C, 58.51; H, 7.37; N, 34.12; found C, 58.30; H, 7.54; N, 34.15.

2,6-Dichloro-9-cyclopentyl-9*H*-purine (**14**) and 2,6-dichloro-7-cyclopentyl-7*H*-purine (**14a**): To a solution of 2,6-dichloropurine (**13**, 1 g, 5.29 mmol) in 5 ml of dry DMF, bromocyclopentane (680 µL, 6.35 mmol) and K_2_CO_3_ (1022 mg, 7.41 mmol) were added and the mixture was allowed to sit at r.t. for 5 days. The volatiles were evaporated, and the residue purified by flash column chromatography, eluting with cHex-EtOAc (90:10 to 70:30). Compounds **14** (530 mg, 2.06 mmol; yield 39%) and **14a** (95 mg, 0.37 mmol; yield 7%) were obtained as white solids.

Compound **14**: m.p.: 117–119 °C. ^1^H-NMR (DMSO-*d_6_*) δ: 1.69 (m, 2H, CH_2_), 1.86 (m, 2H, CH_2_), 2.00 (m, 2H, CH_2_), 2.18 (m, 2H, CH_2_), 4.92 (m, 1H, N-CH), 8.82 (s, 1H, H-8). ESI-MS, positive mode *m*/*z*: 257.9 ([M+H]^+^); 380.1 ([M+Na]^+^). Elemental analysis calcd for C_10_H_10_Cl_2_N_4_: C, 46.71; H, 3.92; N, 21.79; found C, 46.83; H, 3.81; N, 21.69

Compound **14a**: m.p.: 130–133 °C. ^1^H-NMR (DMSO-*d_6_*) δ: 1.67 (m, 2H, CH_2_), 1.85 (m, 2H, CH_2_), 1.99 (m, 2H, CH_2_), 2.14 (m, 2H, CH_2_), 5.01 (m, 1H, N-CH), 8.97 (s, 1H, H-8). Elemental analysis calcd for C_10_H_10_Cl_2_N_4_: C, 46.71; H, 3.92; N, 21.79; found C, 46.78; H, 3.85; N, 21.84.

*N*-((1*H*-benzo[*d*]imidazol-2-yl)methyl)-2-chloro-9-cyclopentyl-9*H*-purin-6-amine (**15**): **14** (150 mg; 0.58 mmol) was reacted with 2-aminomethylbenzimidazole (141 mg; 0.64 mmol) in 5 mL of dry DMF and in the presence of Et_3_N (403 µL; mmol), under a nitrogen atmosphere, at r.t. for 30 h. The mixture was evaporated to dryness and purified by flash column chromatography, eluting with cHex-DCM-MeOH (50:48:2) to obtain **15** (130 mg, 0.35 mmol; yield 60%) as a white powder after crystallization from MeCN. M.p.: 195–197 °C. ^1^H-NMR (DMSO-*d_6_*) δ: 1.71 (m, 2H, CH_2_), 1.87 (m, 2H, CH_2_), 1.97 (m, 2H, CH_2_), 2.15 (m, 2H, CH_2_), 4.82 (m, 1H, N-CH), 4.89 (m, 2H, N^6^-C*H_2_*), 7.13 (bs, 2H, H-Ph), 7.50 (m, 2H, H-Ph), 8.32 (s, 1H, H-8), 8.72 (s, 1H, N^6^H), 12.19 (s, 1H, NH-benzimidazole). ESI-MS, positive mode *m*/*z*: 367.9 ([M+H]^+^); 389.9 ([M+Na]^+^); 756.9 ([2M+Na]^+^). Elemental analysis calcd for C_18_H_18_ClN_7_: C, 58.77; H, 4.93; N, 26.66; found C, 58.72; H, 4.98; N, 26.71.

*N*^6^-((1*H*-benzo[*d*]imidazol-2-yl)methyl)-*N*^2^-benzyl-9-cyclopentyl-9*H*-purine-2,6-diamine (**16**): Compound **15** (100 mg, 0.27 mmol) was suspended in 3 mL of benzylamine with K_2_CO_3_ (112 mg, 0.81 mmol) in an hermetically sealed vial and heated at 100 °C for 48 h. The volatiles were evaporated, H_2_O was added, and the mixture extracted with EtOAc (20 mL X 3). Then, the organic layers were dried over anhydrous Na_2_SO_4_, then filtered and purified by flash column chromatography, eluting with c-Hex-DCM-MeOH (50:48:2). Compound **16** (13 mg, 0.03 mmol; yield 11%) was obtained as a white solid after crystallization with isopropanol. M.p.: 202–204 °C. ^1^H-NMR (DMSO-*d_6_*) δ: 1.64 (m, 2H, CH_2_), 1.83 (m, 2H, CH_2_), 1.94 (m, 2H, CH_2_), 2.03 (m, 2H, CH_2_), 4.39 (m, 2H, N-CH_2_), 4.65 (m, 1H, N-CH), 4.83 (d, *J* = 7.2 Hz, 2H, N^6^-CH_2_), 7.02 (bs, 1H, N^2^H), 7.12 (m, 5H, H-Ph), 7.27 (bs, 2H, H-Ph), 7.41 (bs, 1H, H-8), 7.53 (bs, 1H, N^6^H), 7.79 (bs, 2H, H-Ph), 12.05 (s, 1H, NH-benzimidazole). ESI-MS, positive mode *m*/*z*: 439.1 ([M+H]^+^); 461.3 ([M+Na]^+^). Elemental analysis calcd for C_25_H_26_N_8_: C, 68.47; H, 5.98; N, 25.55; found C, 68.46; H, 5.91; N, 25.62.

*N*^6^-((1*H*-benzo[*d*]imidazol-2-yl)methyl)-9-cyclopentyl-*N*^2^,*N*^2^-dimethyl-9*H*-purine-2,6-diamine (**17**): An excess of liquid dimethylamine was condensed in a sealed vial and cooled with liquid nitrogen. Then, **15** (35 mg, 0.09 mmol) was added and the vial was hermetically closed and heated at 100 °C for 12 h. The volatiles were evaporated, and the residue was purified by flash column chromatography, eluting with DCM-MeOH (97:3). Compound **15** (12 mg, 0.03 mmol; yield 33%) was obtained as a white solid. M.p.: 215–217 °C. ^1^H-NMR (DMSO-*d_6_*) δ: 1.68 (m, 2H, CH_2_), 1.88 (m, 2H, CH_2_), 1.99 (m, 2H, CH_2_), 2.10 (m, 2H, CH_2_), 2.99 (m, 6H, 2xCH_3_), 4.71 (m, 1H, N-CH), 4.86 (d, *J* = 6.9 Hz, 2H, N^6^-CH_2_), 7.10 (m, 2H, H-Ph), 7.40 (m, 2H, H-Ph), 7.52 (m, 1H, N^2^H), 7.82 (m, 2H, H-8 and N^6^H), 12.06 (s, 1H, NH-benzimidazole). ESI-MS, positive mode *m*/*z*: 377.0 ([M+H]^+^); 753.2 ([2M+H]^+^); 399.0 ([M+Na]^+^); 755.2 ([2M+Na]^+^). Elemental analysis calcd for C_20_H_24_N_8_: C, 63.81; H, 6.43; N, 29.77; found C, 63.90; H, 6.42; N, 29.85.

1-Benzyl-3-(2-chloro-9-cyclopentyl-9*H*-purin-6-yl)urea (**18**): Compound **1** (100 mg, 0.42 mmol) was solubilized in 3 mL of dry DMF, then benzyl isocyanate (0.2 mL; 1.68 mmol) and Et_3_N (0.17 mL; 1.26 mmol) were added in a hermetically sealed vial, which was heated to 100 °C for 24 h. The volatiles were evaporated and the mixture purified by flash column chromatography using Isolera Biotage, and eluting with cHex-EtOAc (60:40). Crystallization from EtOH gave **18** (76 mg; 0.21 mmol; yield 49%) as a pure compound. M.p.: 214–215 °C. ^1^H-NMR (DMSO-*d_6_*) δ: 1.70 (m, 2H, CH_2_), 1.87 (m, 2H, CH_2_), 1.97 (m, 2H, CH_2_), 2.17 (m, 2H, CH_2_), 4.49 (d, *J* = 6.2 Hz, 2H, CH_2_Ph), 4.88 (m, 1H, CH), 7.36 (m, 5H, H-Ph), 8.53 (s, 1H, H-8), 9.12 (t, *J* = 7.0 Hz, 1H, NHCH_2_), 10.16 (s, 1H, NHCO). ESI-MS, positive mode *m*/*z*: 370.09 [M+H]^+^, 392.9 [M+Na]^+^. Elemental analysis calcd for C_18_H_19_ClN_6_O: C, 58.30; H, 5.16; N, 22.66; found C, 58.36; H, 5.10; N, 22.59.

Methyl (2-chloro-9-cyclopentyl-9*H*-purin-6-yl)glycinate (**19**): To **14** (200 mg; 0.77 mmol), dissolved in dry THF (5 mL), methyl glycinate (250 mg; 3.08 mmol) was added together with Cs_2_CO_3_ (500 mg; 1.54 mmol) and a catalytic amount of CuI. The mixture was left under a nitrogen atmosphere at r.t. for 6 h. The volatiles were evaporated and the residue, suspended in H_2_O, was extracted with EtOAc (20 mL X 3). The organic layers were dried over anhydrous Na_2_SO_4_, then filtered and evaporated, and the residue purified by flash column chromatography using Isolera Biotage, and eluting with cHex-EtOAc (50:50). Compound **19** (216 mgl, 0.70 mmol; yield 91%) was obtained as a pure solid. M.p.: 144–145 °C. ^1^H-NMR (DMSO-*d_6_*) δ: 1.70 (m, 2H, CH_2_), 1.87 (m, 2H, CH_2_), 1.96 (m, 2H, CH_2_), 2.16 (m, 2H, CH_2_), 3.66 (s, 3H, CH_3_), 4.17 (d, *J* = 7.2 Hz, 2H, C*H*_2_N), 4.80 (m, 1H, CH), 8.30 (s, 1H, H-8), 8.83 (t, *J* = 6.3 Hz, 1H, NH). ESI-MS, positive mode *m*/*z*: 309.8 [M+H]^+^, 331.8 [M+Na]^+^. Elemental analysis calcd for C_13_H_16_ClN_5_O_2_: C, 58.30; H, 5.16; N, 22.66; found C, 58.36; H, 5.10; N, 22.59.

Methyl (2-chloro-9-cyclopentyl-9*H*-purin-6-yl)glycinate (**20**): **19** (50 mg, 0.16 mmol) was suspended in 2 mL of benzylamine. The mixture was left, under a nitrogen atmosphere, at 60 °C for 24 h. The volatiles were evaporated and the mixture purified by a reverse phase column chromatography using Isolera Biotage, and eluting with MeCN-H_2_O (50:50). Compound **20**(46 mg, 0.12 mmol; yield 75%) was obtained as a white solid. M.p.: 161–162 °C. ^1^H-NMR (DMSO-*d_6_*) δ: 1.69 (m, 2H, CH_2_), 1.86 (m, 2H, CH_2_), 1.94 (m, 2H, CH_2_), 2.14 (m, 2H, CH_2_), 4.06 (d, *J* = 7.2 Hz, 2H, CH_2_Ph), 4.30 (d, *J* = 7.0 Hz, 2H, CH_2_CO), 4.80 (m, 1H, CH), 7.28 (m, 5H, H-Ph), 8.28 (s, 1H, H-8), 8.34 (t, *J* = 7.4 Hz,1H, NH), 8.46 (t, 1H, NH). ESI-MS, positive mode *m*/*z*: 384.9 [M+H]^+^, 406.9 [M+Na]^+^, 791.0 [2M+Na]^+^. Elemental analysis calcd for C_19_H_21_ClN_6_O: C, 59.30; H, 5.50; N, 21.84; found C, 59.30; H, 5.45; N, 21.88.

2-((2-Chloro-9-cyclopentyl-9*H*-purin-6-yl)amino)acetonitrile (**21**): To a solution of **14** (130 g, 0.51 mmol) in 5 mL of dry THF, aminoacetonitrile (515 mg, 9.18 mmol) and Et_3_N (0.8 mL, 14.28 mmol) were added. The mixture was allowed to react under stirring at r.t. for 18 h, then the volatiles were evaporated and the residue purified by flash column chromatography, eluting with cHex-EtOAc (60:40). Compound **21**(108 mg, 0.41 mmol; yield 81%) was obtained as a white powder. M.p.: 238–240 °C. ^1^H-NMR (DMSO-*d_6_*) δ: 1.70 (m, 2H, CH_2_), 1.87 (m, 2H, CH_2_), 1.96 (m, 2H, CH_2_), 2.16 (m, 2H, CH_2_), 4.45 (bs, 2H, CH_2_CN), 4.88 (m, 1H, CH), 8.36 (s, 1H, H-8), 8.85 (bs, 1H, NH). ESI-MS, positive mode *m*/*z*: 276.9 [M+H]^+^, 298.9 [M+Na]^+^, 553.1 [2M+H]^+^. Elemental analysis calcd for C_12_H_13_ClN_6_: C, 52.08; H, 4.74; N, 30.37; found C, 52.20; H, 4.62; N, 30.36.

General procedure for the synthesis of 2-chloro-9-cyclopentyl-*N*-(4-methylphenethyl)-9*H*-purin-6-amine (**23**) and 2-chloro-9-cyclopentyl-N-(3-phenylpropyl)-9H-purin-6-amine (**24**): To **14** (150.0 mg, 0.58 mmol), solubilized in MeCN (10 mL), 2-phenylethylamine (169.7 mg, 1.40 mmol) or 3-phenylpropylamine(87 mg, 0.64 mmol) and K_2_CO_3_ (120 mg, 0.87 mmol) were added and the mixture was left under a nitrogen atmosphere at r.t. for 12 h. The volatiles were evaporated and H_2_O was added to the residue, which was extracted with EtOAc (20 mL X 3). The organic layers were dried over anhydrous Na_2_SO_4_, then filtered and purified by flash column chromatography, eluting with cHex-EtOAc (60:40). Compounds **23** (80 mg, 0.23 mmol; yield 40%) and **24** (80 mg, 0.22 mmol; yield 38%) were obtained as white powders.

Compound **23**: m.p. 100–101 °C. ^1^H-NMR (DMSO-*d_6_*): δ 1.72 (m, 2H, CH_2_), 1.89 (m, 2H, CH_2_), 1.97 (m, 2H, CH_2_), 2.15 (m, 2H, CH_2_), 2.90 (t, *J* = 7.2 Hz, 2H, CH_2_Ph), 3.66 (q, *J* = 7.1 Hz, 2H, CH_2_N), 4.77 (m, 1H, N-CH), 7.28 (m, 5H, H-Ph), 8.22 (s, 1H, H-8), 8.29 (brs,1H, NH). ESI-MS, positive mode *m*/*z*: 342.0 [M+H]^+^. Elemental analysis calcd for C_18_H_20_ClN_5_: C, 63.24; H, 5.90; N, 20.49; found C, 63.20; H, 5.98; N, 20.42.

Compound **24**: m.p. 177–179 °C. ^1^H-NMR (DMSO-*d_6_*) δ 1.68 (m, 2H, C*H*_2_CH_2_Ph), 1.72 (m, 2H, CH_2_), 1.88 (m, 2H, CH_2_), 1.95 (m, 2H, CH_2_), 2.14 (m, 2H, CH_2_), 2.64 (t, *J* = 7.0 Hz, 2H, CH_2_Ph), 3.44 (q, *J* = 7.2 Hz, 2H*,* CH_2_N), 4.76 (m, 1H, N-CH), 7.25 (m, 5H, H-Ph), 8.23 (s, 1H, H-8), 8.32 (brs, 1H, NH). ESI-MS, positive mode *m*/*z*: 355.9 [M+H]^+^, 377.9 [M+Na]^+^. Elemental analysis calcd for C_19_H_22_ClN_5_: C, 64.13; H, 6.23; N, 19.68; found C, 64.18; H, 6.20; N, 19.71.

2-Chloro-*N*-(4-chlorophenethyl)-9-cyclopentyl-9*H*-purin-6-amine (**25**): To **14** (150.0 mg, 0.58 mmol), solubilized in 3 mL of dry DMF, 2 (p-chlorophenyl)ethylamine (0.9 mL, 0.64 mmol) and Et_3_N (0.4 mL, 2.9 mmol) were added and the mixture was left at r.t. for 24 h. The volatiles were evaporated and H_2_O was added to the residue, which was extracted with EtOAc (20 mL X 3). The organic layers were dried over anhydrous Na_2_SO_4_, then filtered and purified by flash column chromatography using Isolera Biotage, eluting with cHex-EtOAc (70:30 to 40:60). Compound **25** (122 mg, 0.30 mmol; yield: 52%) was obtained as a white powder. M.p.: 124–125 °C. ^1^H-NMR (DMSO-*d_6_*): δ 1.84 (m, 2H, CH_2_), 1.86 (m, 2H, CH_2_), 1.94 (m, 2H, CH_2_), 2.15 (m, 2H, CH_2_), 2.92 (t, *J* = 7.0 Hz, 2H, CH_2_Ph), 3.64 (m, 2H, CH_2_N), 4.75 (m, 1H, N-CH), 7.31 (m, 4H, H-Ph), 8.23 (s, 1H, H-8), 8.30 (t, *J* = 7.3Hz,1H, NH). ESI-MS, positive mode *m*/*z*: 375.9 [M+H]^+^, 397.8 [M+Na]^+^. Elemental analysis calcd for C_18_H_19_Cl_2_N_5_: C, 57.46; H, 5.09; N, 18.61; found C, 57.41; H, 5.14; N, 18.67.

### 3.2. CK1δ Activity Assays

Procedures reported in the literature were followed to screen the compounds towards truncated CK1δ (Merck Millipore, Merck KGaA, Darmstadt, Germany, recombinant human, GST-tagged, aa 1-294) with the KinaseGlo^®^ kit (Promega, Promega Italy Srl, Milan, Italy) [60]. Luminescent assays were performed in white 96-well plates in a 40 μL final volume with buffer containing 50 mM HEPES (pH 7.5), 1 mM EDTA, 1 mM EGTA, and 15 mM MgCl_2_. In a typical assay, 10 μL of inhibitor solution (desired concentrations were obtained by diluting a starting 10 mM solution in DMSO) and 10 μL of enzyme solution were added to the well, followed by 20 μL of assay buffer containing casein substrate (Sigma-Aldrich, Merck KGaA, Darmstadt, Germany, casein solution from bovine milk, 5% in water) and ATP, at a final concentration of 0.05% and 2 μM, respectively. The final enzyme concentration was 6.5 nM. Compound PF-670462 (IC_50_ = 14 nM) was used as a positive control for CK1δ [61], while a DMSO/buffer solution was used as a negative control. The final DMSO concentration in the reaction mixture did not exceed 1%. After 60 min of incubation at 30 °C, the enzymatic reactions were stopped with the addition of 40 μL of KinaseGlo reagent. The luminescence signal (relative light unit, RLU) was recorded after 10 min at 25 °C using Tecan Infinite M100. First, the residual enzyme activity percentage was determined at 40 μM for each inhibitor with respect to the DMSO/buffer only, and at 10 μM for the compounds showing an enzyme residual activity lower than 50% at 40 μM; subsequently, for the most active compounds, the IC_50_ values were determined using ten different inhibitor concentrations ranging from 100 to 0.026 μM. Data were analyzed using Excel and GraphPad Prism software (version 8.0).

### 3.3. Biological Assays at Human Adenosine Receptors

#### 3.3.1. Cell Culture

Chinese hamster ovary (CHO) cells stably expressing human ARs were grown adherently and maintained in Dulbecco’s Modified Eagles Medium with nutrient mixture F12 (DMEM/F12), supplemented with 10% Fetal Bovine Serum (FBS), 100 U/mL penicillin, 100 µg/mL streptomycin, 2.5 µg/mL amphotericin, 1 mM sodium pyruvate, and 0.1 mg/mL Geneticin (G418) at 37 °C, and aerated with 5% CO_2_: 95% O_2_.

#### 3.3.2. Membrane Preparation

All of the pharmacological methods followed the procedures as described earlier [25]. In brief, the membranes for radioligand binding were prepared from CHO cells stably transfected with human adenosine receptor subtypes through two centrifugations at different speeds. The first low-speed (OKg) centrifugation allowed for the removal of cell fragments and nuclei, while the second, performed at high speed (100,000× *g*), allowed for the precipitation of the crude membrane fractions. The resulting membrane pellet was resuspended in the buffer used for the respective binding experiments, frozen in liquid nitrogen, and stored in aliquots at −80 °C.

#### 3.3.3. Binding Assay

The binding affinity of the novel compounds was evaluated using radioligand competition experiments in CHO cells stably expressing hA_1_AR, hA_2A_AR, hA_3_AR subtypes, as early described [25]. Results were expressed as K_i_ values (dissociation constants), which were calculated with the program GraphPad (GraphPad Software, San Diego, CA, USA). Each concentration was tested three–five times in duplicate and the values given as the mean ± standard error (S.E.). The potency of compounds at the hA_2B_AR (expressed on CHO cells) was determined through a GloSensor cAMP Assay.

#### 3.3.4. Functional Study at Human A_2A_ and A_2B_ Ars

Functional activity was determined as described earlier [34]. Briefly, cells stably expressing both the hA_2A_ or hA_2B_ AR and the plasmid pGloSensor-22F coding for the biosensor were cultured. This biosensor encodes for a genetically modified form of firefly luciferase into which a cAMP-binding protein moiety was inserted. The desiderate cell number was harvested and incubated for 2 h at r.t., with 3% *v*/*v* GloSensor cAMP reagent stock solution, 10% FBS, and 87% CO_2_ independent medium. Cells were dispensed in the wells of a 384-well plate and the reference agonist NECA or the under-study compounds were added at different concentrations. Since the compounds were unable to stimulate cAMP production, they were studied as antagonists. The antagonist profile was evaluated by assessing the ability to counteract an NECA-induced increase in cAMP accumulation. Responses were expressed as a percentage of the maximal relative luminescence units (RLU). Concentration–response curves were fitted by a nonlinear regression using Prism software. The antagonist profile of the compounds was expressed as the IC_50_, which is the concentration of antagonist that produces a 50% inhibition of the agonist effect.

#### 3.3.5. Statistical Analysis

Quantitative data are presented as means ± SE from 3–5 independent experiments. The significance of differences was evaluated using a two-tailed Student’s *t*-test or one-way ANOVA followed by Dunnett’s post-test. Statistical analysis was carried out with GraphPad Prism 8 Software (San Diego, CA, USA). *p* ≤ 0.05 was considered statistically significant [35].

### 3.4. Molecular Modeling Studies

#### 3.4.1. Software Overview

All of the very general molecular modeling operations were executed within the Molecular Operating Environment (MOE) suite (version 2019.01) [62], exploiting an 8 CPU (Intel Xeon E5-1620 3.50 GHz) Linux Workstation, which was also used for the molecular docking calculation. The Supervised Molecular Dynamics (SuMD) simulations were carried out with ACEMD [63] (version 3.3.0), a piece of commercial software based on OpenMM [64] (version 7.4.0). These simulations were executed on a cluster composed of 20 NVIDIA GPUs.

#### 3.4.2. Protein Preparation

For the reasons already mentioned at the beginning of this paragraph, the experimental protein structures chosen for the molecular modeling operations were the ones coming from the complexes with the PDB codes 4TN6, 7LD4, and 5K2C for CK1δ, A_1_AR, and A_2A_AR, respectively. For these protein–ligand systems, their structure was downloaded and imported into the main window of the MOE suite, and then underwent a proper preparation procedure for computational handling. This process involved, first, the MOE Structure preparation tool, which was used to give each amino acid sidechain the proper conformational state based on the occupancy. After that, the Protonate 3D application was exploited to assign each amino acid its appropriate protonation state at pH 7.4. In the end, the added hydrogen atoms were energetically minimized under the AMBER10:EHT [65] force field implemented in MOE. As already said, the structure for the A_3_AR protein was created by homology modeling using the structure of A_1_AR (which was reported to be more reliable for this task in a recent paper by Margiotta et al. [42]) as a template. More specifically, the approach adopted was the one named “ligand-based homology modeling” [66], which relies on the fact that, if the computational model of a protein is created based on a template in which a ligand is crystallized into the binding site, the model will also keep the “imprint” of such a molecule, resembling, to a higher extent, a proper crystallographic protein–ligand complex, and thus, being more suitable for molecular docking calculations. From a practical point of view, we executed the “ligand-based homology modeling” by exploiting the dedicated MOE homology modeling tool, taking into account the adenosine molecule present in the orthosteric binding site of the crystal structure with PDB code 7LD4, the A_1_AR experimental complex used as the template.

#### 3.4.3. Ligand Preparation

The 18 adenine small molecules composing the database under analysis were all prepared by exploiting the tools of the QUACPAC [67] package from the OpenEye suite. First, the dominant tautomeric state was selected for each molecule using the tautomers application; then, the MMFF-based three-dimensional coordinates for the compounds were generated with Omega. The partial charges for each compound were then calculated with the MolCharge tool, using AM1-BCC as the method [68]. Finally, the dominant protonation state at pH 7.4 was selected using the FixpKa application.

#### 3.4.4. Molecular Docking

As already mentioned in the previous paragraphs, the molecular docking calculations were executed with the program PLANTS, generating 25 poses for each compound in the orthosteric binding site of the target, which was selected by using the center of mass of the crystallographic ligand as the center of a sphere of 15 Å radius. The scoring function selected was PLANTS_CHEMPLP_.

#### 3.4.5. System Setup for Supervised Molecular Dynamics (SuMD) Simulations

The compound **17** (the “dual anta-inhibitor”) and compound **21** recognition mechanisms towards both CK1δ and A_2A_AR were further characterized through Supervised Molecular Dynamics (SuMD) simulations (see next paragraph). Concerning CK1δ, simulations were carried out according to the AMBER 14 force field [65]: each preparation passage was performed either through VMD 1.9.2 [69] or through the appropriate package from ambertools22 [70]. At first, the ligand was placed at 30 Å from the nearest protein atom, enough to allow the ligand to explore its conformational freedom before reaching the binding site. Ligand partial charges were assigned through the AM1-BCC method [68]. Ligand parameters were assigned through the General Amber Force Field 2 (GAFF2) [71]. Then, each protein–ligand system was solvated in a TIP3P [72] water box, ensuring a 15 Å distance between the box border and the nearest protein atom. The simulation box was then neutralized through the addition of a proper number of Na^+^ and Cl^−^ ions until a salt concentration of 0.154 M was reached. Before production, each system was subjected to a two-stage equilibration protocol. At first, 500 steps of energy minimization through the conjugate gradient algorithm were carried out to eliminate clashes and bad contacts that could destabilize the system. Then, a first equilibration simulation was carried out for 0.1 ns in the canonical ensemble (NVT), imposing harmonic positional restraints of 5 kcal mol^−1^Å^−2^ on both the protein and the ligand atoms. Finally, a second equilibration simulation was performed for 0.5 ns in the isobaric-isothermal ensemble (NPT), maintaining the harmonic positional restrain of 5 kcal mol^−1^Å^−2^ on the ligand and protein backbone only. Regarding A_2A_, simulations were carried out according to the CHARMM force field [73]: each preparation passage was carried out through the appropriate tools of VMD 1.9.3. Once again, the ligand was placed at 30 Å from the nearest protein atom, enough to allow the ligand to explore its conformational freedom before reaching the binding site. The conserved sodium ions located in the transmembrane site, along with the three coordination water molecules, were retained in the generation of the system, in agreement with previous studies from our laboratory, which showed how, despite not exerting any influence on the docking calculations [74], it can have an important effect on the outcome of molecular dynamics simulations involving antagonists [59]. The system was described using parameters from the CHARMM36 force field (protein, lipids, ions, and water molecules), while the ligand parameters were retrieved from Paramchem, a web front-end for the CGenFF force field [75]. At first, the protein−ligand system was explicitly solvated in a cubic TIP3P water box, ensuring a distance of 15 Å between the box borders and any protein atom. Then, the system charge was neutralized by the addition of sodium and chlorine ions until a physiological concentration (0.154 M) was reached. Finally, the receptor was embedded in a lipid bilayer consisting of phosphatidylcholine (POPC) units. Before production, each system was subjected to a four-stage equilibration protocol. Initially, 1500 steps of energy minimization, using the conjugate gradient method, were performed to remove clashes and bad contacts within the system. The first three equilibration MD simulations were carried out in the isothermal−isobaric ensemble (NPT), while the fourth and final one was performed in the isothermal ensemble (NVT). The first equilibration stage consisted of a 5 ns simulation with 5 kcal mol^−1^ Å^−2^ harmonic positional constraints applied on each receptor, ligand, and membrane atom. The second equilibration stage consisted of a 10 ns simulation with the same constraints applied only to each protein, ligand, and phosphorus atom. The third equilibration stage consisted of a 5 ns simulation with the same constraints applied only to the protein backbone and the ligand atoms. Finally, a 10 ns equilibration MD simulation was performed without any constraints applied to the system. For both the equilibration and productive simulations, the integration timestep was set to 2 fs and the temperature was set to 310 K through a Langevin thermostat (friction coefficient = 0.1 ps^−1^) [76]. For simulations in the NPT ensemble, the pressure was maintained at 1.0 atm exploiting a Monte Carlo barostat [77]. The M-SHAKE [78] algorithm was employed to constrain the length of bonds involving hydrogen atoms, and the particle mesh Ewald (PME) [79] was exploited to compute the electrostatic interactions (grid length = 1 Å). Finally, a 9.0 Å cutoff was applied to long-term interactions.

#### 3.4.6. Supervised Molecular Dynamics (SuMD) Simulations

Supervised Molecular Dynamics [55,58] is an enhanced-sampling molecular dynamics protocol that allows for the investigation of molecular recognition processes between biomolecular entities in a nanosecond timescale, contrary to the microseconds that are usually required for classic, unsupervised, molecular dynamics simulations. A SuMD simulation is composed of a series of short, classic molecular dynamics simulations, defined as “SuMD-steps”. Each “SuMD-step” is carried out in the canonical ensemble (NVT), exploiting the Langevin thermostat to maintain the system at the physiological temperature (310 K), using an integration timestep of two femtoseconds, the M-SHAKE algorithm to constrain bonds involving hydrogen atoms, the particle-mesh Ewald (PME) method for the calculation of electrostatic interactions, and a cutoff of 9.0 Å for the computation of the Lennard–Jones interaction. The duration of each “SuMD-step” is user-defined, based on the ligand’s complexity: in this case, as previously performed in other similar works when investigating binding pathways involving small organic molecules as ligands, the length of a “SuMD-step” was set to be 600 ps. At the end of each “SuMD-step”, the distance between the center of the mass of the ligand and the user-defined binding site is computed for each simulation snapshot. These distance values are then interpolated by a linear function, which is fed to a taboo-like algorithm: if the slope of the line is negative, indicating that the ligand is approaching the binding site throughout the considered simulation window, the “SuMD-step” is considered productive and, therefore, retained for the generation of the final trajectory; the next “SuMD-step” is then instantiated using the final state of said step. On the contrary, if the slope value is positive, indicating that the ligand is distancing itself from the binding site all along the considered simulation windows, the “SuMD-step” is discarded and the simulation is restarted from the previous productive “SuMD-step”, randomly reassigning the velocities through the Langevin thermostat. The geometric supervision upon the binding process is switched off after the distance between the two centers of mass reaches below a user-defined threshold value (5 Å, in this case): after this checkpoint has been reached, the simulation continues for other 30 steps of classic, unsupervised, molecular dynamics simulation. The code to run SuMD simulations is written in the Python programming language and exploits the ACEMD3 engine [63], which is based on the OpenMM7 Python library [64], to perform molecular dynamics simulations, while the geometric supervision of the trajectories is carried out through the ProDy library [80]. The code is available at https://github.com/molecularmodelingsection/SuMD accessed on 12 January 2023.

#### 3.4.7. SuMD Trajectories Analyses

The SuMD trajectories generated, as described in the previous paragraph, were analyzed to depict the pivotal interaction determinants that steer the investigated protein–ligand recognition process, using the same protocols already utilized in previous scientific work from our laboratory [45]. At first, the system was wrapped into an image of itself under periodic boundary conditions. Then, each trajectory frame was aligned on the protein backbone, taking as a reference the first trajectory frame. Both these operations were carried out through the appropriate tools of Visual Molecular Dynamics (VMD) 1.9.2. Each pre-processed trajectory was then analyzed, making use of an in-house Python script, available at github.com/molecularmodelingsection/SuMD-analyzer. Particularly, a per-residue interaction energy decomposition was carried out by exploiting the NAMDEnergy plugin v1.4 for VMD [81]. The 25 most contacted receptor residues throughout the trajectory were considered for this analysis. The results of this analysis were then plotted onto an interaction energy heatmap through the Seaborn Python package. In the plot, the simulation time (in nanoseconds) is reported on the horizontal axis, and the receptor residue is reported on the vertical axis, while the interaction energy value (in kcal mol^−1^) is color plotted according to a divergent colormap, where blue indicates negative (thus attractive) values and red indicates positives (thus repulsive) values. The same script used for the generation of the maps was also exploited for the generation of the trajectory videos provided as Supplementary Materials.

## 4. Conclusions

By screening an in-house chemical library of A_2A_AR antagonists endowed with an adenine structure and tested as CK1δ inhibitors, the 2-chloro-9-cyclopentyladenine (**1**) was selected for further modifications. Hence, a series of 9-cyclopentyladenine derivatives substituted at the 2/*N*^6^- or 2/8-positions were synthesized and evaluated for their ability to inhibit the CK1δ enzyme and to bind ARs. In particular, the *N*^6^-acetonitrile-2-chloro-9-cyclopentyladenine (**21**; IC_50_ = 0.36 μM) resulted in the most active CK1δ inhibitor of the series, while the 2-chloro-8-thiophenyl-9-cyclopentyladenine (**9**; KiA_1_ = 0.053 μM and KiA_3_ = 0.017 μM) and the 2-chloro-8-furyl-9-cyclopentyladenine (**8**; KiA_2A_ = 0.007 μM) showed the best affinity at the A_1_/A_3_ and A_2A_ ARs. Although the most potent enzyme inhibitor showed a moderate affinity for ARs and vice versa, some compounds, that we called “dual anta-inhibitors”, were found to possess enzyme inhibitory activities with IC_50_ values in the sub-μM range and AR affinities with nM Kis. Hence, the 2-chloro-*N*^6^-(2-phenylethyl)-9-cyclopentyladenine (**23**; KiA_3_ = 0.151 μM, IC_50_ = 0.66 μM) and the 2-chloro-*N*^6^-(3-phenylpropyl)-9-cyclopentyladenine (**24**; KiA_1_ = 0.692 μM, IC_50_ = 0.73 μM) resulted in dual anta-inhibitors of CK1δ and A_1_ and A_3_ ARs, respectively, while the 9-cyclopentyl-2-dimethylaminoadenine (**12**; KiA_2A_ = 0.123 μM, IC_50_ = 1.75 μM) and 9-cyclopentyl-2-dimethylamino-*N*^6^-methyl-(2-benzimidazolyl)adenine (**17**; KiA_2A_ = 0.076 μM, IC_50_ = 0.59 μM) were dual anta-inhibitors of CK1δ and A_2A_ARs. Computational studies performed with molecular docking and dynamics provided a simulation of the ligand–target interactions for these compounds in protein targets, with interpretations of the possible impacts of the compound substituents on ligand recognition. Compound **17**, endowed with the best balance of the two activities, represents the first ever reported “dual anta-inhibitor” of the A_2A_AR and CK1δ enzyme, and is the leading compound for potential therapeutic agents with synergistic neuroprotective and antitumor effects.

## Figures and Tables

**Table 1:** Enzymatic inhibition activity of compounds **1**, **5**, **6**, **8**–**12**, **15**–**21**, **23**–**25** tested at 40 and 10 μM (IC_50_, μM).

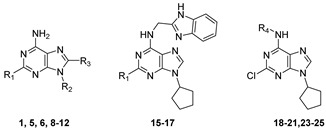							
**Cmp**	**R_1_**	**R_2_**	**R_3_**	**R_4_**	**% Residual Activity at 40 μM ± sd**	**% Residual Activity at 10 μM ± sd**	**IC_50_ (μM)**
**1**	Cl	cC_5_H_9_	H		0.70 ± 0.4	13 ± 7.4	5.20 ± 1.2
**5**	Cl	cC_4_H_7_	H		43 ± 3.2	37 ± 3.8	5.25 ± 0.9
**6**	Cl	cC_6_H_11_	H		30 ± 0.2	52 ± 3.0	>10
**8**	Cl	cC_5_H_9_	2-furyl		86 ± 1.6	n.d.	>40
**9**	Cl	cC_5_H_9_	2-thienyl		90 ± 4.6	n.d.	>40
**10**	PhCH_2_NH	cC_5_H_9_	H		30 ± 4.1	48 ± 3.5	5.30 ± 1.0
**11**	Ph(CH_2_)_2_NH	cC_5_H_9_	H		88 ± 2.9	n.d.	>40
**12**	(CH_3_)_2_N	cC_5_H_9_	H		19 ± 9.4	27 ± 4.0	1.75 ± 0.2
**15**	Cl				22 ± 13	24 ± 14	1.53 ± 0.4
**16**	PhCH_2_NH				46 ± 31	26 ± 7.6	4.76 ± 0.3
**17**	(CH_3_)_2_N				22 ± 1.0	34 ± 5.0	0.59 ± 0.2
**18**				CONHCH_2_Ph	29 ± 3.9	38 ± 6.0	3.37 ± 2.5
**19**				CH_2_COOCH_3_	44 ± 2.6	23 ± 2.4	2.55 ± 0.2
**20**				CH_2_CONHCH_2_Ph	29 ± 12	32 ± 16	1.87 ± 0.2
**21**				CH_2_CN	25 ± 9.6	13 ± 24	0.36 ± 0.1
**23**				(CH_2_)_2_Ph	14 ± 9.7	27 ± 20	0.66 ± 0.2
**24**				(CH_2_)_3_Ph	11 ± 19	26 ± 6.7	0.73 ± 0.1
**25**				(CH_2_)_2_Ph-4Cl	14 ± 4.3	31 ± 12	1.69 ± 1.0

**Table 2:** Binding data at the A_1_, A_2A_, and A_3_ ARs of compounds **1**, **5**, **6**, **8**–**12**, **15**–**21**, **23**–**25** at human ARs stably transfected in CHO cells (Ki, **μ**M).

Cmp	R_1_	R_2_	R_3_	R_4_	KiA_1_ (μM) ^a^	KiA_2A_ (μM) ^b^	KiA_3_ (μM) ^c^	IC_50_ (μM) ^d^
**1**	Cl	cC_5_H_9_	H		1.893 ± 0.160	0.465 ± 0.129	0.631 ± 0.062	5.20
**5**	Cl	cC_4_H_7_	H		1.533 ± 0.265	0.558 ± 0.151	7.224 ± 1874	5.25
**6**	Cl	cC_6_H_11_	H		n.d. ^e^	n.d.	n.d.	>10
**8**	Cl	cC_5_H_9_	2-furyl		0.118 ± 0.010	0.007 ± 0.00001	0.140 ± 0.035	>40
**9**	Cl	cC_5_H_9_	2-thienyl		0.053 ± 0.009	0.036 ± 0.009	0.017 ± 0.002	>40
**10**	PhCH_2_NH	cC_5_H_9_	H		3.957 ± 0.800	0.157 ± 0.040	0.298 ± 0.047	5.30
**11**	Ph(CH_2_)_2_NH	cC_5_H_9_	H		0.580 ± 0.046	0.012 ± 0.003	0.049 ± 0.008	>40
**12**	(CH_3_)_2_N	cC_5_H_9_	H		3.339 ± 0.658	0.123 ± 0.002	2.878 ± 0.695	1.75
**15**	Cl				>30	3.257 ± 0.626	1.138 ± 0.004	1.53
**16**	PhCH_2_NH				>30	0.718 ± 0.149	0.432 ± 0.025	4.76
**17**	(CH_3_)_2_N				2.903 ± 0.648	0.076 ± 0.017	1.146 ± 0.107	0.59
**18**				CONHCH_2_Ph	>30	>30	>30	3.37
**19**				CH_2_COOCH_3_	20.675 ± 1.865	13.460 ± 0.980	14.770 ± 3.010	2.55
**20**				CH_2_CONHCH_2_Ph	>30	>30	3.374 ± 205	1.87
**21**				CH_2_CN	8.657 ± 1.524	3.478 ± 0.312	5.957 ± 1.021	0.36
**23**				(CH_2_)_2_Ph	1.336 ± 0.279	1.174 ± 0.037	0.151 ± 0.015	0.66
**24**				(CH_2_)_3_Ph	0.692 ± 0.088	3.335 ± 0.749	1.275 ± 0.289	0.73
**25**				(CH_2_)_2_Ph-4Cl	10.812 ± 2.039	2.655 ± 0.381	2.106 ± 515	1.69

^a^ Displacement of specific [^3^H]CCPA binding in membranes from CHO cells, stably transfected with human recombinant A_1_AR. ^b^ Displacement of specific [^3^H]NECA binding in membranes from CHO cells, stably transfected with human recombinant A_2A_AR. ^c^ Displacement of specific [^3^H]HEMADO binding in membranes from CHO cells, stably transfected with human recombinant A_3_AR. Data (*n* = 3–5) are expressed as means ± standard errors. ^d^ IC_50_ values are referred to CK1δ inhibition. ^e^ Not determined.

## Data Availability

Date sharing is contained in this article.

## Supplementary Materials

The following supporting information can be downloaded at: https://www.mdpi.com/article/10.3390/ph16020167/s1, “Video_S1.mp4”, “Video_S2.mp4”, “Video_S3.mp4”, and “Video_S4.mp4”.
